# Synthesis of Aminobisphosphinates through a Cascade Reaction between Hypophosphorous Acid and Bis(trimethylsilyl)imidates Mediated by ZnI_2_

**DOI:** 10.3390/molecules28176226

**Published:** 2023-08-24

**Authors:** Nouha Ayadi, Aurélie Descamps, Thibaut Legigan, Jade Dussart-Gautheret, Maelle Monteil, Evelyne Migianu-Griffoni, Taïcir Ben Ayed, Julia Deschamp, Marc Lecouvey

**Affiliations:** 1 Department of Chemistry, Université Sorbonne Paris Nord, CSPBAT UMR CNRS 7244, 1 Rue de Chablis, F-93000 Bobigny, France; ayadinouha9@gmail.com (N.A.); descamps.aurelie12@gmail.com (A.D.); jade92.dussart@gmail.com (J.D.-G.); maelle.monteil@univ-paris13.fr (M.M.); migianu@univ-paris13.fr (E.M.-G.); 2 Department of Chemistry, Université de Carthage-INSAT—Eco-Chimie Lab (LR21ES02), Centre Urbain Nord B.P.N. 676, Tunis 1080, Tunisia; taicir.mzidbenayed@insat.ucar.tn

**Keywords:** bisphosphinates, phosphonite, Lewis acid, methodological development

## Abstract

Among phosphorylated derivatives, phosphinates occupy a prominent place due to their ability to be bioisosteres of phosphates and carboxylates. These properties imply the necessity to develop efficient methodologies leading to phosphinate scaffolds. In recent years, our team has explored the nucleophilic potential of silylated phosphonite towards various electrophiles. In this paper, we propose to extend our study to other electrophiles. We describe here the implementation of a cascade reaction between (trimethylsilyl)imidates and hypophosphorous acid mediated by a Lewis acid allowing the synthesis of aminomethylenebisphosphinate derivatives. The present study focuses on methodological development including a careful NMR monitoring of the cascade reaction. The optimized conditions were successfully applied to various aliphatic and aromatic substituted (trimethylsilyl)imidates, leading to the corresponding AMBPi in moderate to good yields.

## 1. Introduction

The synthesis of phosphorylated molecules still represents a major challenge for organic chemists to propose new drugs [1,2,3,4]. Among them, phosphinate derivatives (R^2^R^3^PO_2_R^1^) have gained attention in medicinal chemistry for their potential as bioactive compounds and drug candidates thanks to their ability to mimic the function of phosphates or carboxylates. Indeed, the presence of a P-C bond imparts chemical stability against hydrolysis, whether it occurs through chemical or enzymatic processes [2,4].

**Figure 1:**
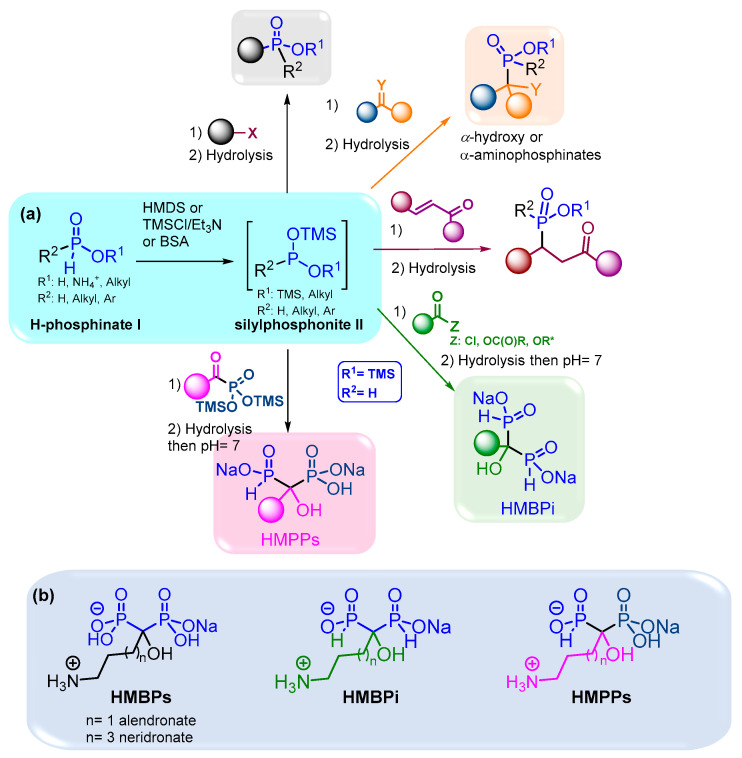
(**a**) Synthesis and uses of silylated phosphonite **II** in the presence of various electrophiles; (**b**) structures of HMBPs and their analogues in bisphosphonate and phosphinylphosphonate series. * Refers to activated esters.

Hence, the development of efficient methodologies is crucial to access phosphorylated scaffolds. The formation of the P-C bond can be managed via several pathways such as transition metal catalysis, radical reactions and nucleophilic additions or substitutions [5]. Among these methods, the use of silylated phosphonite **II** represents a versatile tool that operates smoothly and thus is compatible with functionalized molecules. Moreover, it is easily accessible through a reaction between *H*-phosphinate **I** and a silylated agent like HMDS, TMSCl or bis(trimethylsilyl)acetamide (BSA) depicted in Figure 1. The sila-Arbuzov reaction of silylated phosphonites **II** on alkyl halides as electrophiles can provide substituted alkyl phosphinates.

Aldehydes, ketones and imines can also undergo nucleophilic attack from silylated phosphonites via the Abramov reaction to give various α-hydroxy- and α-aminophosphinates, respectively. In addition, Michael addition on α,β-unsaturated ketones can selectively take place to form functionalized substituted phosphinates [6]. Our group has contributed to the use of the simplest silylated phosphonite: bis(trimethylsilyl)phosphonite **II** (R^1^ = TMS, R^2^ = H: BTSP), obtained starting from hypophosphorous acid (H_3_PO_2_) and BSA as a silylating agent. First, we have demonstrated that only two equivalents of BSA were required to fully transform H_3_PO_2_ into BTSP, despite large excesses of silylated agents being previously employed in the literature [6]. Then, the subsequent addition on aldehydes and ketones provided various α-hydroxyphosphinates as sodium salts in good to excellent yields [7].

Moreover, we also performed the successive double nucleophilic addition of BTSP onto trivalent electrophiles as acyl chlorides, which enabled the formation of hydroxymethylenebisphosphinates (HMBPi) via silylated α-ketophosphinates in good to excellent yields and short reaction times [8,9]. Thereafter, this easily handled methodology was successfully used on other trivalent electrophiles, like anhydrides and activated esters, which led to more functionalized HMBPi derivatives in good yields [10].

This method was subsequently transposed to synthesize hydroxymethylene(phosphinyl)phosphonate derivatives (HMPPs), which consisted of adding BTSP to in situ pre-formed α-ketophosphonates starting from trimethylphosphite and acyl chlorides. This one-pot procedure allowed the preparation of original HMPPs, in which no purification of intermediate species was required (Figure 1a) [11].

Moreover, these methodologies were applied to the synthesis of aminoalkyl-substituted HMBPi and HMPPs, which are analogues of the hydroxymethylenebisphosphonates (HMBPs) currently used in clinics to treat bone diseases such as osteoporosis, solid tumor metastases or myeloma bone disease [12,13,14,15,16,17,18,19]. Moreover, HMBPs have shown interesting antitumor properties on in vitro and in vivo models of soft tissue primary tumors. As a result, the antiproliferative activities of these newly synthesized HMBPi and HMPPs have been evaluated on various cancer cell lines and encouraging results have been obtained, especially on A549 cells (Figure 1b) [11].

**Figure 2:**
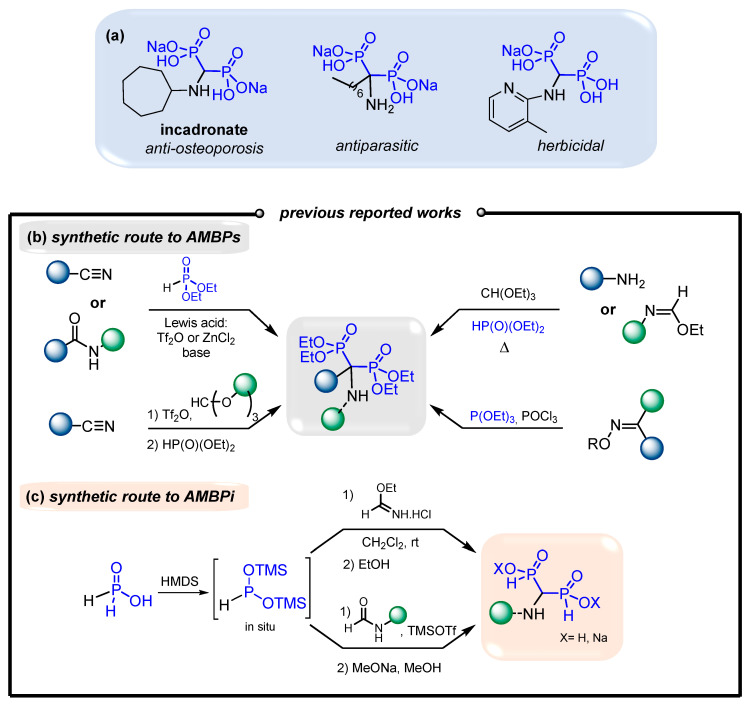
(**a**) Representative bioactive AMBPs. (**b**) Synthetic pathways to AMBPs. (**c**) Synthetic pathways to AMBPi.

Additionally, several α-aminomethylenebisphosphonates (AMBPs) exhibit biological activities that include antiparasitic [20,21], antibacterial [22], herbicidal [23,24] and bone-resorption-inhibitory [14,25] activities (Figure 2a). The access to AMBPs is well documented in the literature [26]. Indeed, several approaches present the double phosphonylation of amides and nitriles mediated by various Lewis acids [27,28,29,30], a three-component reaction of amines with orthoformate and phosphites [31,32,33,34,35,36,37,38,39,40], and a Beckmann transposition of oximes in the presence of phosphites [41,42] (Figure 2b). Alternatively, only limited examples were reported for the synthesis of aminomethylenebisphosphinates (AMBPi) and their biological activities remain unknown to date [43,44,45]. Here, the strategy usually consists of adding in situ pre-formed BTSP onto ethyl formimidate hydrochloride or onto substituted amides in the presence of TMSOTf, respectively (Figure 2c). In this case, only a few *N*-substituted aminomethylenebisphosphinates (AMBPi) were synthesized.

As a result of these works, our team has decided to pursue exploring the nucleophilic potential of BTSP towards less-reactive trivalent electrophiles such as nitriles.

In this case, aminomethylenebisphosphinate (AMBPi) scaffolds will be formed through the successive double addition of BTSP onto nitriles. However, the lack of nitrile reactivity should require the use of a Lewis acid, as was demonstrated for the AMBP series.

**Figure 3:**
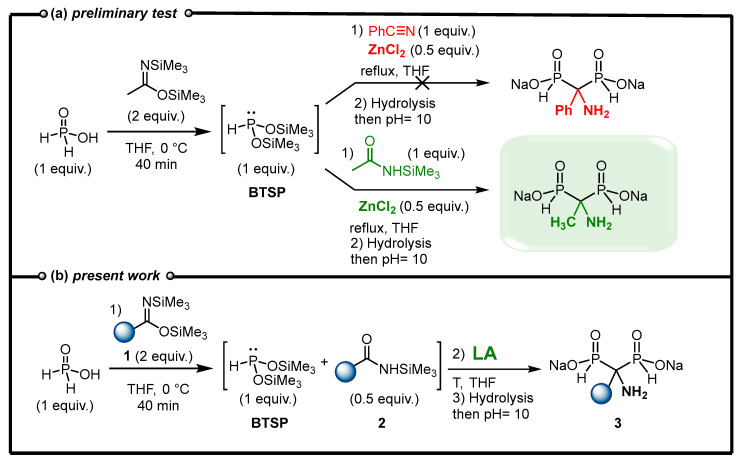
(**a**) Preliminary test on the addition of BTSP to nitrile. (**b**) New methodology leading to AMBPi **3**.

Our initial experiment consisted of the in situ formation of BTSP via silylation of H_3_PO_2_ in the presence of BSA in THF followed by the addition of benzonitrile and ZnCl_2_ as a Lewis acid (Figure 3a). Finally, the reaction mixture was stirred under reflux as no conversion was observed at room temperature. The reaction evolution was monitored via ^31^P and ^31^P{^1^H} NMR experiments. After refluxing for 6 h, the complete conversion of BTSP was observed and an AMBPi derivative was obtained after methanolysis. However, the careful analysis of the ^1^H and ^13^C spectra and mass spectroscopy indicates the formation of an α-aminomethylenebisphosphinate including a methyl substituent instead of the expected phenyl group. Consequently, the reaction did not occur on nitrile but on the *N*-silylacetamide generated during the silylation step in the presence of bis(trimethylsilyl)acetamide, which is in accordance with some reported works previously mentioned [30]. *N*-Silylacetamide appears to be a better electrophile than benzonitrile towards the attack of the nucleophilic BTSP.

In view of this unexpected result, we decided to explore the feasibility of developing a cascade reaction in which bis(trimethylsilyl)imidate **1** could silylate H_3_PO_2_ to simultaneously generate nucleophilic BTSP and an electrophilic *N*-silylamide **2** (Figure 3b). Subsequently, and in the presence of a Lewis acid, these products could react together to enable the formation of AMBPi derivatives. Herein, we present our endeavors to develop an efficient cascade process promoted by a Lewis acid to furnish α-aminomethylenebisphosphinates **3** (AMBPi).

## 2. Results and Discussion

### 2.1. Synthesis of Bis(silyl)imidates ***1***

**Scheme 1:**
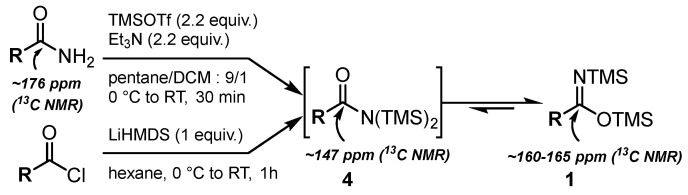
Synthesis of *N*,*O*-bis(silyl)imidates **1**.

First, we focused on the synthesis of *N*,*O*-bis(trimethylsilyl)imidates **1** [46,47], which could be achieved via the silylation of amides [48,49] or by adding LiHMDS to acyl chlorides [46,50,51,52,53] (Scheme 1).

In both cases, the reaction allowed us to form *N*,*N*-bis(silyl)amides **4**, which instantly tautomerized to the more stable *N*,*O*-bis(trimethylsilyl)imidates **1** [46,52]. Indeed, the reaction monitoring via ^13^C NMR enabled us to only detect the quaternary carbon of **1** at ~160–165 ppm. The signal at ~147 ppm corresponding to *N*,*N*-bis(silyl)amides **4** was only observed when 2.2 equivalents of TMSOTf/Et_3_N were sequentially added in two portions to the corresponding acetamide. In our study, the reaction between amides and TMSOTf/Et_3_N was selected due to its ease of implementation and higher efficiency.

### 2.2. Optimization of the Reaction between N,O-Bis(trimethylsilyl)imidates and Phosphorous Acid Mediated by Lewis Acid

The reaction was first carried out between hypophosphorous acid and commercially available *N*,*O*-bis(trimethylsilyl)acetamide **1a** (R = Me) (Table 1). The silylation was monitored via ^31^P NMR and was completed after 40 min at 0 °C.

Thereafter, various Lewis acids were screened for the second reaction between trimethylsilylacetamide **2a** and BTSP (Table 1, entries 1–4). In the presence of zinc halides, AMBPi **3a** was similarly obtained in good conversions and isolated yields after purification (Table 1, entries 1, 2). However, it was noted that the reaction rate was higher with ZnI*_2_* than with ZnCl_2_, as the former allowed the reaction to completed after only 1.5 h, whereas the latter took 18 h. When TMSOTf was used as a Lewis acid, the reaction was able to proceed at 0 °C after only 30 min and furnished AMBPi **3a** in 75% yield (Table 1, entry 3). In contrast, no conversion was observed in the presence of BF_3_·OEt_2_, regardless of the temperature and reaction time (Table 1, entry 4).

Then, the same reactions were performed with freshly prepared *N*,*O*-bis(trimethylsilyl)acetamide **1a** (R = Me) in the presence of ZnX_2_ or TMSOTf (Table 1: entries 5–7 versus 1–3). In these cases, the reactions provided the same results independently of the Lewis acids as expected.

To explore the potential range of the reaction, additional *N*,*O*-bis(trimethylsilyl)imidates **1b** (R = Pr) and **1c** (R = Ph) were initially combined with H_3_PO_2_, and the resulting blend was subsequently subjected to various Lewis acids (Table 1, entries 8–13).

Concerning the use of *N*,*O*-bis(trimethylsilyl)butanimidate **1b**, the silylation of H_3_PO_2_ was completed after 40 min at 0 °C. The consequent double addition of BTSP to the corresponding silylamide **2b** successfully ensued in the presence of ZnX_2_ to furnish the expected AMBPi **3b** after methanolysis and purification (Table 1, entries 8, 9). As previously observed, the reaction rate was higher for ZnI_2_ than for ZnCl_2_. However, we noted a dramatic drop in the conversion into **2b** in the presence of TMSOTf, as a major disproportionation of BTSP was observed (Table 1, entry 10).

Upon investigating the reactivity of aromatic bis(trimethylsilyl)amide (Table 1, entries 11–13), it was revealed that among the various Lewis acids tested, zinc iodide uniquely mediated the attack of BTSP onto **2c**, leading to the proper formation of AMBPi **3c** (Table 1, entry 12). Indeed, no reaction took place in the presence of TMSOTf (Table 1, entry 13); moreover, the use of zinc chloride resulted in the formation of α-aminophosphinate **5c** and a major disproportionation of BTSP into silylated phosphorus derivatives (Table 1, entry 11). Furthermore, the reactivity of zinc chloride seems inadequate to promote the sila-Arbuzov reaction. Additionally, AMBPi **3c** may not be stable enough and seems to lead to the formation of **5c**, as previously described in the literature [43]. Finally, the optimization of the cascade reaction showed the potent use of various commercially available and freshly prepared aliphatic and aromatic *N*,*O*-bis(trimethylsilyl)imidates **1a**–**c** as silylating agents. In addition, the Lewis acid screening highlighted zinc iodide as the best compromise in terms of reactivity and reaction time.

### 2.3. NMR Monitoring and Purification Details

**Figure 4:**
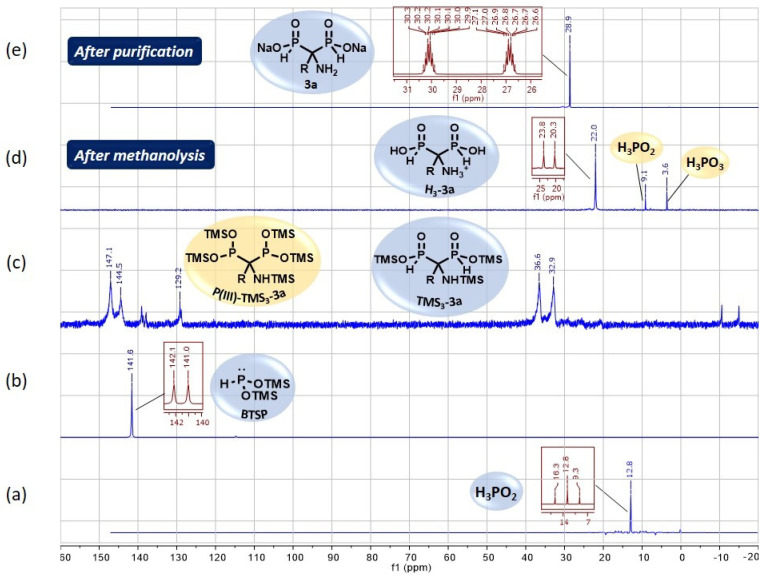
(**a**–**e**) ^31^P (dark red) and{^1^H}^31^P (blue) NMR monitoring of the optimized cascade reaction between phosphorous acid and *N*,*O*-bis(trimethylsilylacetamide **1a**.

As mentioned earlier, ^31^P and ^31^P {^1^H} NMR experiments are routinely performed to follow the course of reactions implying phosphorus derivatives. Figure 4 displays the optimized cascade reaction monitoring of the various phosphorus intermediate species.

The rapid silylation of H_3_PO_2_ was observed through the disappearance of its signal at 12.8 ppm for the benefit of a new signal at 141.6 ppm in the trivalent phosphorus region that confirmed the formation of BTSP as expected (Figure 4, spectra (b) versus (a)).

After refluxing for 1.5 h, the NMR monitoring indicated the complete conversion of BTSP through its missing signal at 141.6 ppm and the appearance of several peaks in the P(III)/P(V) regions (142–147 ppm and 33–36 ppm) related to **P(III)-TMS_3_-3a** and **TMS_3_-3a**, respectively (Figure 4, spectra (c) versus (b)). After methanolysis, a major signal remained at 22.0 ppm, which matches the acidic form **H_3_-3a** of AMBPi **3a** (Figure 4, spectra (d) versus (c)). It was noted that small amounts of H_3_PO_2_ and H_3_PO_3_ were also generated. The pH adjustment to 10 produced AMBPi **3a** as a disodium salt and concomitantly caused the partial precipitation of the zinc salt, which was eliminated via centrifugation. Then, successive washes with ethyl acetate (with 0–10% ethanol) and methanol enabled us to recover the excess amide, and to discard both NaH_2_PO_2_ and Na_2_HPO_3_, respectively. The residual zinc salts were removed thanks to a cation-exchange resin.

The effectiveness of zinc elimination was verified by ICP-AES analysis. Finally, the disodium salt of AMBPi **3a** was isolated after lyophilization as a pure form (Figure 4, spectra (e) versus (d)).

### 2.4. Viability Considerations of the Cascade Reaction

**Scheme 2:**
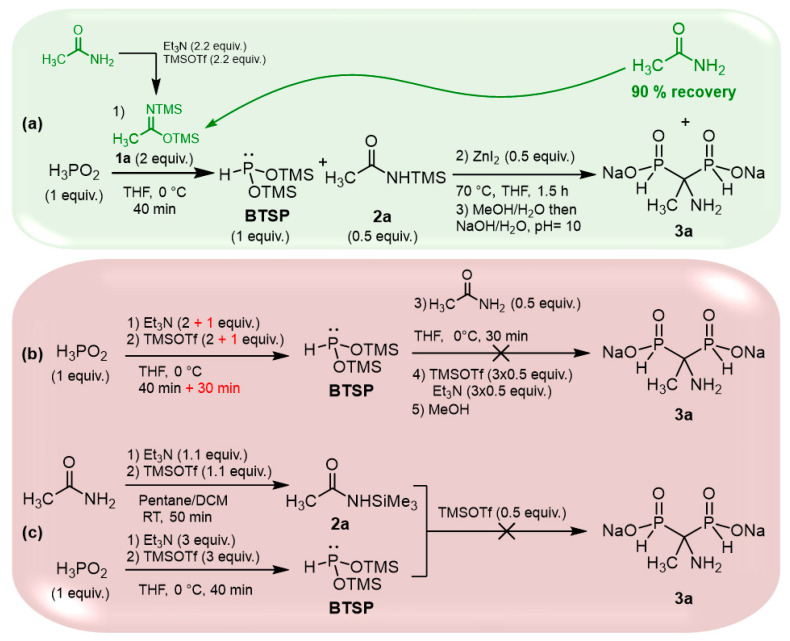
Complementary tests to validate the cascade reaction.

The cascade reaction appears to be an interesting and straightforward method for accessing AMBPi scaffolds; however, 2 equivalents of silylimidates **1** are required for the silylation step, but only a 0.5 equivalent of the silylamide generated in the first step participates in the sila-Arbuzov reaction. To overcome this significant drawback, we focused on retrieving the excess of the amides initially used for the formation of the silylimidates (Scheme 2a).

Several attempts enabled us to finally recover up to 90% of the resulting amides during the purification procedure, which could be reused for the same reaction.

We also wondered if the reaction could proceed via a direct silylation of H_3_PO_2_ in the presence of Et_3_N/TMSOTf followed by the addition of an amide in the presence of TMSOTf. Here, TMSOTf will both play the role of a silylating agent and a Lewis acid (Scheme 2b). First, hypophosphorous acid and two equivalents of triethylamine/TMSOTf were mixed at 0 °C.

Unfortunately, the silylation was partial even after an extended period. To achieve completion, an additional equivalent of Et_3_N/TMSOTf was added, leading to a 30 min reaction time. However, this approach resulted in a significant amount of HP(O)(OTMS)_2_ due to the oxidation of BTSP. In the second step, acetamide and TMSOTf were subsequently introduced at 0 °C. Unfortunately, no AMBPi derivative was formed, and the reaction only resulted in the disproportionation of BTSP, giving hypophosphorous and phosphorous acids.

As a final attempt, we independently synthesized **2a** and BTSP in the presence of triethylamine and TMSOTf, which were subsequently mixed (Scheme 2c). Alas, the reaction did not occur under these conditions.

These assays validated the viability of the cascade reaction we proposed. Moreover, the excess of amide can be successfully recovered, thus limiting its impact on the reaction implementation.

### 2.5. Scope of the Cascade Reaction

**Scheme 3:**
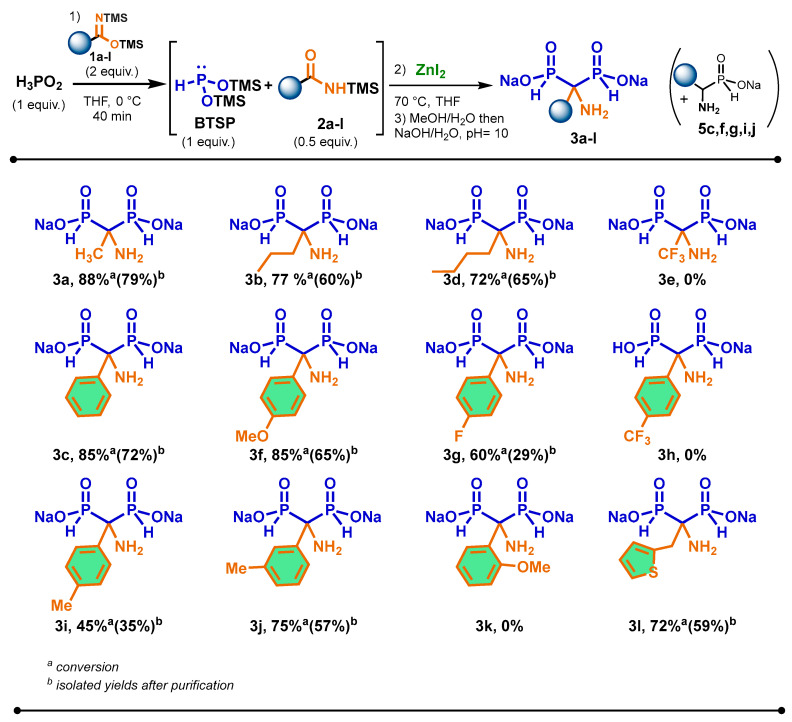
Scope of the cascade reaction between various bis(trimethylsilyl)imidates **2a**–**l** and hypophosphorous acid in the presence of zinc iodide.

The scope of the cascade reaction was carried out in the presence of various prepared aliphatic and aromatic bis(trimethylsilyl)imidates **1a**–**l** amd hypophosphorous acid and zinc iodide as Lewis acids (Scheme 3). As a general trend, all imidates enabled the promotion of the silylation of H_3_PO_2_ into BTSP efficiently.

We were pleased to observe that the reaction was successful with aliphatic imidates bearing longer-chain **1b** and **1d**. In these cases, the corresponding AMBPi **3b** and **3d** were obtained in good conversions and isolated yields after purification. Although bis(trimethylsilyl)trifluoroacetimidate **1e** can properly promote the silylation of H_3_PO_2_, the sila-Arbuzov reaction did not happen. Only the oxidation of BTSP was noted. 

Concerning the use of aromatic bis(trimethylsilyl)imidate derivatives, the reactivity of *para*-substituted aromatic imidate derivatives was evaluated under the previous optimized conditions. The substitution at the *para*-position by a methoxy group had little influence on the course of the reaction, which produced AMBPi **3f** in similar yield to **3c**. When the reaction was performed with a *para*-substituted methyl moiety on silylamide **2i**, the yield for the formation of **3i** surprisingly decreased. However, the *meta*-substituted methyl aromatic amide **2j** properly underwent the sila-Arbuzov reaction with good conversion and isolated yield of **3j**. The reaction was also carried out with electrowithdrawing *para*-fluoro- and *para*-trifluoromethyl-substituted groups on amides **2g** and **2h**. Although the conversion into **3g** reached 60%, the isolated yield dropped to 29% due to its oxidation during the purification. Moreover, **3h** was not produced as only the disproportionation of BTSP took place.

The introduction of an *ortho*-substituted methyl group and a heteroaromatic moiety on the aromatic imidates **2k** and **2l** was also considered. In these cases, only AMBPi **3l** was generated in good conversion and isolated yield, probably due to the steric hindrance of substrate **2k**.

It was noted that α-aminophosphinates **5c**,**f**,**g**,**i**,**j** were detected after methanolysis. According to the NMR spectra, these compounds represented 10 to 15% (^31^P NMR) of crude products. This observation could justify the lower yields obtained for these AMBPi **3c**,**f**,**g**,**i**,**j**.

Even if the results were moderate for some substituted aromatic AMBPi, this cascade reaction represents the first example of an AMBPi which displays alkyl or aromatic groups at the methylene carbon on α-aminomethylenebisphosphinates.

### 2.6. Plausible Mechanism of the Cascade Reaction

**Scheme 4:**
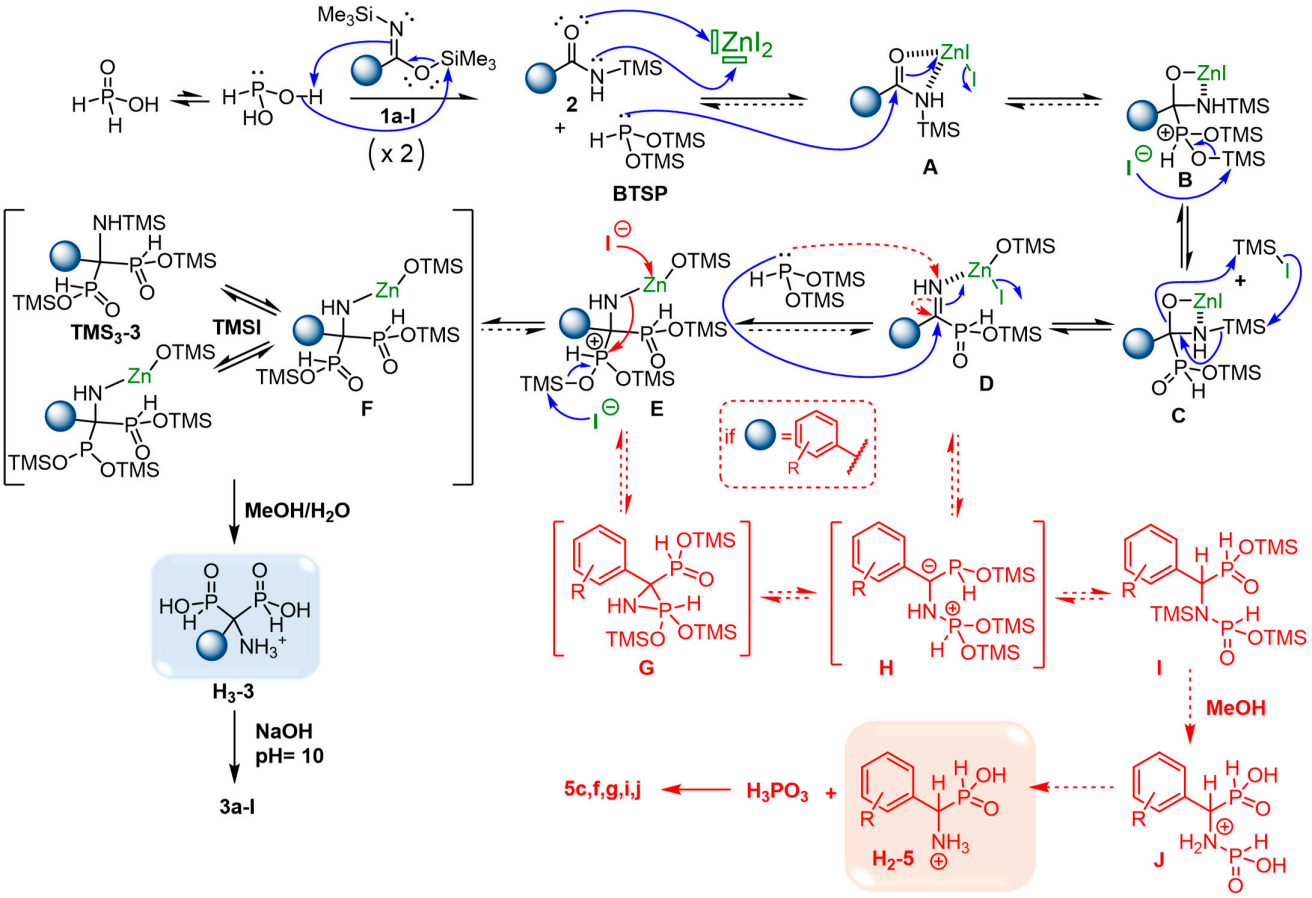
Postulated mechanism for the formation of AMBPi **3a**–**l** and α-aminophosphinate **5c**,**f**,**g**,**i**,**j**.

In view of our past and present results, the following mechanism depicted in Scheme 4 could be proposed to explain the formation of AMBPi **3a**–**l** and α-aminophosphinates **5c**,**f**,**g**,**i**,**j** [9,10].

First, the existing equilibrium between hypophoshorous acid and its trivalent form could be disrupted by trapping the latter as the silylated phosphonite BTSP in the presence of silylimidate **1a**–**l**. Subsequently, the generated silylamide **2a**–**l,** activated by zinc iodide, could undergo attack by BTSP resulting in the formation of a phosphonium intermediate **B**. Following this, a series of trans-silylation steps could lead to the silylated α-iminophosphinate **D**. A second attack of BTSP on intermediate **D** could give the second phosphonium derivative **E**, which participates in various trans-silylation equilibria. These equilibria are supported by the presence of multiple trivalent and pentavalent species during the ^31^P{^1^H} NMR reaction monitoring. Finally, the methanolysis step yields the acidic form **H_3_-3**, subsequently leading to the expected AMBPi **3a**–**l** through pH adjustment to 10.

The formation of the aromatic α-aminophosphinate byproducts **5c**,**f**,**g**,**i**,**j** could be explained by the relative instability of AMBPi, which may rapidly degrade in α-aminophosphinate and phosphorous acid as already reported in the literature [43]. However, the side products **5c**,**f**,**g**,**i**,**j** were only observed in the presence of aromatic and/or steric-hindered silylamides **2c**,**f**,**g**,**i**,**j**, which implies another possible degradation pathway during the reaction process. In our past studies on the synthesis of HMBPi, phosphinylphosphonate byproducts resulting from transposition reactions were identified when the reaction was carried out with electrowithdrawing substituted aromatic and/or steric-hindered acyl chlorides and BTSP. Here, we proposed similar possible side routes starting from α-iminophosphinate **D** or/and the phosphonium derivative **E**. Indeed, the phosphonylated derivatives **D** and **E** could evolve into a benzylic stabilized anion **H** resulting from the attack of BTSP on the nitrogen atom of **D** or/and the ring opening of phosphaziridine **G**, respectively. The anion **H** could subsequently move towards the silylated phosphinylphosphonamidate **I**, which afforded compound **H_2_-5** due to the hydrolysis of **J**. Further investigations might be undertaken to corroborate this postulated mechanism.

## 3. Materials and Methods

### 3.1. General Informations

Reagents were purchased from usual commercial suppliers (Sigma-Aldrich, Alfa Aesar, Acros Organics, Saint-Quentin-Fallavier, France) and used as delivered. Triethylamine was distillated and stored over KOH under argon. Extra-dry grade solvents (Acros Organics, St. Louis, MO, USA) were used. *N*,*O*-*bis*(trimethylsilyl)acetamide (BSA) was purchased from Alfa Aesar, Karlsruhe, DE, USA (batch number: 10186753). Anhydrous H_3_PO_2_ was dehydrated from a commercially available aqueous solution of H_3_PO_2_ (50% *w*/*w*) according to the procedure reported by Montchamp et al. [54]. Reactions requiring inert conditions were carried out in flame-dried glassware under an argon atmosphere. The solvents were degassed via argon bubbling for 30 min.

NMR spectra were recorded at 20 °C on a Bruker Avance-III-400 spectrometer, Billerica, MA, USA (^1^H: 400 MHz, ^13^C: 101 MHz, ^31^P: 162 MHz, ^19^F: 377 MHz). Chemical shifts (δ) were given in ppm, the number of protons (n) for a given resonance was indicated by n H and coupling constants *J* in Hz. ^1^H NMR spectra were calibrated on a non-deuterated solvent residual peak (H_2_O: 4.79 ppm), while H_3_PO_4_ (85% in water) was used as an external standard for ^31^P NMR. The following abbreviations were used for ^1^H, ^13^C, ^31^P and ^19^F NMR spectra to indicate the signal multiplicity: s (singlet), d (doublet), t (triplet), dd (doublet of doublets), dm (doublet of multiplet), m (multiplet), dq (doublet of quartets) and ddq (doublet of doublets of quartets). All ^13^C NMR spectra were measured with ^1^H decoupling while ^31^P and ^19^F NMR spectra were measured with ^1^H coupling and ^1^H decoupling. ^1^H experiments with water presaturation were performed with D_1_ = 2 s and 128 scans. The reactions were followed by ^31^P and ^31^P{^1^H} NMR experiments (the spectra were recorded without lock and shims). All NMR peak assignments were performed thanks to 2D NMR COSY, HMQC and HMBC experiments. High-resolution mass spectra (HRMS) were obtained on a Bruker maXis mass spectrometer in negative (ESI^-^) mode (ESI) via the “Fédération de Recherche” ICOA/CBM (FR2708) platform. MS analyses were performed using a QTOF Impact HD mass spectrometer equipped with an electrospray (ESI) ion source (Bruker Daltonics, Billerica, MA, USA). The instrument was operated in the negative mode with an ESI source on a Q-TOF mass spectrometer with an accuracy tolerance of 2 ppm. Samples were diluted with acetonitrile and water (15:85) and were analyzed via mass spectrometry in continuous infusion using a syringe pump at 200 µL/min. The mass profiles obtained via ESI-MS were analyzed using Data Analysis software (Bruker Daltonics). ICP-AES analyses were performed via “plateforme Analytiques des Inorganiques” IPHC UMR7178 on Varian 720ES, Palo Alto, CA, USA.

### 3.2. General Procedure for the Cascade Synthesis of Aminomethylenebisphosphinates ***3a***–***l***

To a dry and argon-flushed 100 mL three-necked flask, equipped with a thermometer, an argon inlet and a septum, the corresponding amide **1a**–**l** (15.00 mmol, 1.00 equiv.), anhydrous pentane (34.00 mL), anhydrous dichloromethane (1.50 mL) and triethylamine (33.00 mmol, 5.58 mL, 2.20 equiv.) were successively introduced. Trimethylsilyltrifloromethanesulfonate (33.00 mmol, 5.73 mL, 2.20 equiv.) was added dropwise at 0 °C and the mixture was stirred for 30 min at room temperature. The lower phase obtained during the process was eliminated. Then, the solvent was evaporated under reduced pressure. The imidates **2a**–**l** were used in the next step without further purification.

To another dry and argonflushed 25 mL three -necked flask equipped with a thermometer, a reflux condenser with an argon inlet and a septum, anhydrous hypophosphorous acid (5.00 mmol, 0.330 g, 0.50 equiv.) and anhydrous tetrahydrofuran (1.00 mL) were added under an argon atmosphere. The synthesized imidates **2a**–**l** (10.00 mmol, 2.00 equiv.) were added dropwise at 0 °C and the mixture was stirred for 40 min. The reaction conversion was monitored via ^31^P NMR. A solution of zinc iodide (2.50 mmol, 0.750 g, 0.50 equiv.) in anhydrous tetrahydrofuran (4.00 mL) was added dropwise at 0 °C and the mixture was stirred under reflux conditions. The reaction conversion was also monitored via ^31^P NMR upon completion. Then, anhydrous methanol (3.00 mL) was added dropwise at 0 °C followed by water (0.5 mL). The solvents were evaporated, and the crude compound was dissolved in minimum of water (2.00 mL) and an aqueous solution of sodium hydroxide (0.50 M, 8.00 mL) was added carefully to adjust the pH to 10.00. The mixture was centrifugated to partially discard precipitated zinc salts. The filtrate was then washed with ethyl acetate (5 × 5.00 mL) (with 0–10% ethanol) and methanol (10 × 2.00 mL) to eliminate the excess of amides **1a**–**l** and phosphorous acid, respectively. In addition, a cation-exchange resin was used to eliminate the residual zinc salts. Finally, the solution was lyophilized to afford the pure AMBPi **3** as a disodium salt.

### 3.3. Spectral Data of Aminomethylenebisphosphinates ***3a***–***l***

**1-aminoethane-1,1-bis(*H*-phosphinate) disodium salts 3a**. White powder; 425 mg, 79% yield. ^31^P {^1^H} NMR (162 MHz, D_2_O) δ 28.5 (s). ^31^P NMR (162 MHz, D_2_O) δ 28.5 (dm, ^1^*J_P-H_* = 524.9 Hz). ^1^H NMR (400 MHz, D_2_O) δ 6.80 (dt, ^1^*J_P-H_* = 525.3 Hz, ^2^*J* = 11.7 Hz, 2H), 1.19 (t, ^2^*J_P-H_* = 15.8 Hz, 3H). ^13^C NMR (101 MHz, D_2_O) δ 52.2 (t, ^1^*J_P-C_* = 89.4 Hz), 15.0. MS (ESI^-^) *m*/*z* 171.99 [M − H]^−^, 193.97 [M − 2H + Na]^−^, 153.98 [M − H-H_2_O]^−^. HRMS (ESI^-^) *m*/*z*: [M − H]^−^. Calcd. for [C_2_H_8_NO_4_P_2_]: 171.9934; found: 171.9934.
**1-amino-1-propylmethane-1,1-bis(*H*-phosphinate) disodium salts 3b.** White powder; 359 mg, 60% yield. ^31^P {^1^H} NMR (162 MHz, D_2_O) δ 28.1 (s). ^31^P NMR (162 MHz, D_2_O) δ 28.1 (dp, ^1^*J_P-H_* = 523.6 Hz, ^2^*J* = 13.4 Hz). ^1^H NMR (400 MHz, D_2_O) δ 6.84 (dt, ^1^*J_P-H_* = 523.5 Hz, ^2^*J* = 12.1 Hz, 2H), 1.72–1.55 (m, 2H), 1.54–1.39 (m, 2H), 0.88 (t, *J* = 7.2 Hz, 3H). ^13^C NMR (101 MHz, D_2_O) δ 55.3 (t, ^1^*J_P-C_* = 89.0 Hz, 33.1, 16,6 (t, ^2^*J_P-C_* = 6.8 Hz), 14.3. MS (ESI^-^) *m*/*z* 200.02 [M − H]^−^, 222.00 [M − 2H + Na]^−^, 182.01 [M − H-H_2_O]^−^, 134.04 [M − H-H_3_PO_2_]^−^. HRMS (ESI^-^) *m*/*z*: [M − H]^−^. Calcd. for [C_4_H_12_NO_4_P_2_]: 200.0247; found: 200.0247.
**1-amino-1-phenylmethane-1,1-bis(*H*-phosphinate) disodium salts 3c**. White powder; 500 mg, 72% yield. ^31^P {^1^H} NMR (162 MHz, D_2_O) δ 26.8 (s). ^31^P NMR (162 MHz, D_2_O) δ 26.8 (dt, ^1^*J_P-H_* = 538.6 Hz, *J* = 12.1 Hz). ^1^H NMR (400 MHz, D_2_O) δ 7.43 (d, ^3^*J_P-H_* = 8.1 Hz, 2H), 7.32 (t, *^4^J_P-H_* = 7.6 Hz, 2H), 7.23 (t, ^1^*J_P-H_* = 7.6 Hz, 1H), 6.87 (dt, ^1^*J_P-H_* = 539.0 Hz, ^2^*J* = 10.3 Hz, 2H). ^13^C NMR (101 MHz, D_2_O) δ 164.0, 128.5, 127.0, 126.0, 60.5 (t, ^1^*J_P-C_* = 86.0 Hz. MS (ESI^-^) *m*/*z* 234.00 [M − H]^−^, 215.99 [M − H-H_2_O]^−^, 170.04 [M − H-HPO_2_]^−^. HRMS (ESI^-^) *m*/*z*: [M − H]^−^. Calcd. for [C_7_H_10_NO_4_P_2_]: 234.0090; found: 234.0090.
**1-amino-1-butylmethane-1,1-bis(*H*-phosphinate) disodium salts 3d**. White powder; 417 mg, 65% yield. ^31^P {^1^H} NMR (162 MHz, D_2_O) δ 27.9 (s). ^31^P NMR (162 MHz, D_2_O) δ 27.9 (dp, ^1^*J_P-H_* = 523.8 Hz, ^2^*J* = 13.2 Hz). ^1^H NMR (400 MHz, D_2_O) δ 6.85 (dt, ^1^*J_P-H_* = 524.3 Hz, ^2^*J* = 11.9 Hz, 2H), 1.73–1.62 (m, 2H), 1.48–1.40 (m, 2H), 1.28 (hex., *J* = 7.3 Hz, 2H), 0.86 (t, *J* = 7.3 Hz, 3H). ^13^C NMR (101 MHz, D_2_O) δ 55.2 (t, ^1^*J_P-C_* = 88.6 Hz), 30.45 (C_2_), 25.1 (t, ^3^*J_P-C_* = 6.7 Hz), 23.0, 13.1. MS (ESI^-^) *m*/*z* 214.04 [M − H]^−^, 236.02 [M − 2H + Na]^−^, 196.03 [M − H-H_2_O]^−^. HRMS (ESI^-^) *m*/*z*: [M − H]^−^. Calcd. for [C_5_H_14_NO_4_P_2_]: 214.0403; found: 214.0403.
**1-amino-1-(4-methoxyphenyl)methane-1,1-bis(*H*-phosphinate) disodium salts 3f**. White powder; 0.483 mg, 65% yield. ^31^P {^1^H} NMR (162 MHz, D_2_O) δ 26.8 (s). ^31^P NMR (162 MHz, D_2_O) δ 26.8 (dt, ^1^*J_P-H_* = 538.7 Hz, ^2^*J* = 12.4 Hz). ^1^H NMR (400 MHz, D_2_O) 7.47 (d, ^3^*J* = 8.9 Hz, 2H), 7.02 (d, ^3^*J_P-H_* = 8.5 Hz, 2H), δ 6.95 (dt, ^1^*J_P-H_* = 537.9 Hz, ^2^*J*= 11.1 Hz, 2H), 3.81 (s, 3H). ^13^C NMR (101 MHz, D_2_O) δ 157.8 (t, *J* = 2.4 Hz), 128.2, 127.4 (t, ^3^*J_P-C_* = 4.9 Hz), 114.0, 59.8 (t, ^1^*J_P-C_* = 86.8 Hz), 55.3. MS (ESI^-^) *m*/*z* 264.02 [M − H]^−^, 286.00 [M − 2H + Na]^−^, 246.01 [M − H-H_2_O]^−^, 200.05 [M − H-HPO_2_]^−^. HRMS (ESI^-^) *m*/*z*: [M-H]^−^. Calcd. for [C_8_H_12_NO_5_P_2_]: 264.0196; found: 200.0204.
**1-amino-1-(4-fluorophenyl)methane-1,1-bis(*H*-phosphinate) disodium salts 3g**. White powder; 220 mg, 29% yield. ^31^P {^1^H} NMR (162 MHz, D_2_O) δ 26.4 (s). ^31^P NMR (162 MHz, D_2_O) δ 26.4 (dt, ^1^*J_P-H_* = 538.6 Hz, *J* = 11.1 Hz). ^19^P NMR (377 MHz, D_2_O) δ 116.8 (m). ^1^H NMR (400 MHz, D_2_O) δ 7.57–7.46 (m, 2H), 7.14 (t, *^4^J_P-H_* = 8.8 Hz, 2H), 6.96 (dt, ^1^*J_P-H_* = 537.4 Hz, ^1^*J_P-H_* = 10.8 Hz, 2H). ^13^C NMR (101 MHz, D_2_O) δ 160.7 (dt, ^1^*J_C-F_* = 243.2 Hz, *^4^J_P-C_* = 2.8 Hz), 131.6–131.5 (m), 127.8–127.7 (m), 115.2, 115.0, 60.0 (t, ^1^*J_P-C_* = 86.0 Hz). MS (ESI^-^) *m*/*z* 252.00 [M − H]^−^, 273.98 [M − 2H + Na]^−^, 233.99 [M − H-H_2_O]^−^, 188.03 [M − H-HPO_2_]^−^. HRMS (ESI^-^) *m*/*z*: [M − H]^−^. Calcd. for [C_7_H_9_FNO_4_P_2_]: 251.9996; found: 251.9996.
**1-amino-1-(4-tolyl)methane-1,1-bis(*H*-phosphinate) disodium salts 3i.** White powder; 250 mg, 35% yield. ^31^P {^1^H} NMR (162 MHz, D_2_O) δ 26.8 (s). ^31^P NMR (162 MHz, D_2_O) δ 26.8 (dt, ^1^*J_P-H_* = 539.8 Hz, *J* = 12.0 Hz). ^1^H NMR (400 MHz, D_2_O) δ 7.46–7.40 (m, 2H), 7.26–7.22 (m, 2H), 6.96 (dt, ^1^*J_P-H_* = 538.2 Hz, ^2^*J* = 10.8 Hz, 2H), 2.30 (s, 3H). ^13^C NMR (101 MHz, D_2_O) δ 137.0, 132.6, 129.1, 126.05 (t, ^3^*J_P-C_* = 4.8 Hz), 60.2 (t, ^1^*J_P-C_* = 86.5 Hz), 20.1. MS (ESI^-^) *m*/*z* 248.02 [M − H]^−^, 270.00 [M − H-H_2_O]^−^, 184.05 [M − H-HPO_2_]^−^. HRMS (ESI^-^) *m*/*z*: [M − H]^−^. Calcd. for [C_8_H_12_FNO_4_P_2_]: 248.0247; found: 248.0247.
**1-amino-1-(3-tolyl)phenyl)methane-1,1-bis(*H*-phosphinate) disodium salts 3j.** White powder; 400 mg, 57% yield. ^31^P {^1^H} NMR (162 MHz, D_2_O) δ 26.8 (s). ^31^P NMR (162 MHz, D_2_O) δ 26.8 (dt, ^1^*J_P-H_* = 542.3 Hz, *J*= 11.5 Hz). ^1^H NMR (400 MHz, D_2_O) δ 7.36 (s, 1H), 7.30−7.29 (m, 2H), 7.15 (s, 1H), 6.96 (dt, ^1^*J_P-H_* = 538.9 Hz, ^2^*J* = 11.8 Hz, 2H), 2.32 (s, 3H). ^13^C NMR (101 MHz, D_2_O) δ 138.4, 135.8 (t, *^4^J_P-C_* = 2.3 Hz), 128.4, 127.6, 126.7 (t, ^3^*J_P-C_* = 4.9 Hz), 123.0 (t, ^3^*J_P-C_* = 5.0 Hz), 60.5 (t, ^1^*J_P-C_* = 86.1 Hz), 20.7. MS (ESI^-^) *m*/*z* 248.02 [M − H]^−^, 270.01 [M − 2H + Na]^−^, 230.01 [M − H-H_2_O]^−^, 184.05 [M − H-HPO_2_]^−^. HRMS (ESI^-^) *m*/*z*: [M − H]^−^. Calcd. for [C_8_H_12_NO_4_P_2_]: 248.0247; found: 248.0247.
**1-amino-1-(2-thienyl)ethane-1,1-bis(*H*-phosphinate) disodium salts 3l.** White powder; 437 mg, 59% yield. ^31^P {^1^H} NMR (162 MHz, D_2_O) δ 26.5 (s). ^31^P NMR (162 MHz, D_2_O) δ 26.5 (dm, ^1^*J_P-H_* = 531.6 Hz). ^1^H NMR (400 MHz, D_2_O) δ 7.28–7.27 (m, 1H, H_6_), 6.99–6.97 (m, 2H), 6.83 (dt, ^1^*J_P-H_* = 530.6 Hz, ^2^*J* = 11.8 Hz, 2H), 3.26 (t, *J* = 12.7 Hz, 2H). ^13^C NMR (101 MHz, D_2_O) δ 136.9 (t, ^3^*J_P-C_* = 9.1 Hz), 128.5, 126.9, 125.1, 55.1 (t, ^1^*J_P-C_* = 89.3 Hz), 29.8. MS (ESI^-^) *m*/*z* 253.98 [M − H]^−^, 275.96 [M − 2H + Na]^−^, 253.97 [M − H-H_2_O]^−^. HRMS (ESI^-^) *m*/*z*: [M − H]^−^. Calcd. for [C_6_H_10_NO_4_P_2_S]: 253.9811; found: 253.9811.

## 4. Conclusions

In this study, we have established a cascade reaction involving the silylation of hypophosphorous acid with a *N*,*O*-bis(trimethylsilyl)imidate, leading to the formation of bis(trimethylsilyl)phosphonite (BTSP) and a *N*-silylamide. The latter can subsequently undergo nucleophilic attack of BTSP through a sila-Arbuzov reaction, which is mediated by zinc iodide as a Lewis acid. This approach relies on an unexpected result as our initial attempt was to investigate the reactivity of nitriles in the presence of BTSP and a Lewis acid. We present a detailed methodology to propose a novel means to access AMBPi scaffolds, which have been understudied in the literature. The screening of Lewis acids has highlighted zinc iodide as the best promoter for the sila-Arbuzov reaction. Consequently, we successfully synthesized various AMBPi **3a**–**l** in moderate to good yields. Better results were obtained in aliphatic series and will enable us to extend this method to more functionalized AMBPi, analogous to aminomethylenebisphosphonates, which have demonstrated relevant biological activities.

## Figures and Tables

**Table 1:** Optimizations of reaction parameters.

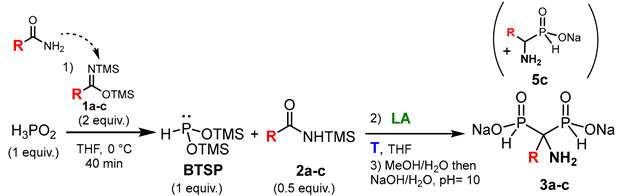						
Entry	2a–c	R	LA	T, °C	Time, Hour	3a–c/5c, Yield (%) ^1^
Entry 1	**2a**	Me ^5^	ZnCl_2_	70	18	**3a**, 90 ^2^ (75) ^3^
Entry 2	**2a**	Me ^5^	ZnI_2_	70	1.5	**3a**, 88 ^2^ (83) ^3^
Entry 3	**2a**	Me ^5^	TMSOTf	0	0.5	**3a**, 90 ^2^ (75) ^3^
Entry 4	**2a**	Me ^5^	BF_3_·OEt_2_	0–70	18	-
Entry 5	**2a**	Me ^6^	ZnCl_2_	70	15	**3a**, 90 ^2^ (75) ^3^
Entry 6	**2a**	Me ^6^	ZnI_2_	70	1.5	**3a**, 88 ^2^ (79) ^3^
Entry 7	**2a**	Me ^6^	TMSOTf	0	0.5	**3a**, 90 ^2^ (75) ^3^
Entry 8	**2b**	Pr	ZnCl_2_	70	18	**3b**, 77 ^2^ (60) ^3^
Entry 9	**2b**	Pr	ZnI_2_	70	2	**3b**, 77 ^2^ (60) ^3^
Entry 10	**2b**	Pr	TMSOTf	0	0.5	**3b**, 6 ^4^
Entry 11	**2c**	Ph	ZnCl_2_	70	18	**5c**, 15 ^4^
Entry 12	**2c**	Ph	ZnI_2_	70	1	**3c**, 85 ^2^ (72) ^3^
Entry 13	**2c**	Ph	TMSOTf	0	0.5	-

^1^ The reaction evolution was monitored via ^31^P NMR. ^2^ The conversions were determined via ^31^P NMR after methanolysis. ^3^ Isolated yields after purification. ^4^ Proportion determined via ^31^P NMR after methanolysis in the crude mixture. ^5^ Commercially available BSA was used. ^6^ BSA was synthesized according to the procedure in the literature.

## Data Availability

The data presented in this study are available in the article or in the Supplementary Materials.

## Supplementary Materials

The following supporting information can be downloaded at: https://www.mdpi.com/article/10.3390/molecules28176226/s1. ^1^H-, ^13^C- and ^31^P- spectra of AMBPi are available online.

## Sample Availability

Samples of compounds **3a**–**l** are available from the authors.

## References

[1] Horsman G.P., Zechel D.L. (2017). Phosphonate Biochemistry. Chem. Rev..

[2] Virieux D., Volle J.N., Bakalara N., Pirat J.L. (2015). Synthesis and biological applications of phosphinates and derivatives. Top. Curr. Chem..

[3] Pradere U., Garnier-Amblard E.C., Coats S.J., Amblard F., Schinazi R.F. (2014). Synthesis of nucleoside phosphate and phosphonate prodrugs. Chem. Rev..

[4] Yu H., Yang H., Shi E., Tang W. (2020). Development and Clinical Application of Phosphorus-Containing Drugs. Med. Drug. Discov..

[5] Montchamp J.-L. (2019). Challenges and solutions in phosphinate chemistry. Pure Appl. Chem..

[6] Montchamp J.-L. (2005). Recent advances in phosphorus–carbon bond formation: Synthesis of H-phosphinic acid derivatives from hypophosphorous compounds. J. Organomet. Chem..

[7] Dussart J., Deschamp J., Monteil M., Gager O., Migianu-Griffoni E., Lecouvey M. (2019). A General Protocol for the Synthesis of H --Hydroxyphosphinates. Synthesis.

[8] Guedeney N., Dussart J., Deschamp J., Ouechtati M., Migianu-Griffoni E., Lecouvey M. (2019). A convenient one-pot synthesis of 1-hydroxymethylene-1,1-bisphosphinic acids. Phosphorus Sulfur Silicon Relat. Elem..

[9] Dussart J., Guedeney N., Deschamp J., Monteil M., Gager O., Legigan T., Migianu-Griffoni E., Lecouvey M. (2018). A convenient synthetic route towards H-bisphosphinates. Org. Biomol. Chem..

[10] Dussart-Gautheret J., Deschamp J., Monteil M., Gager O., Legigan T., Migianu-Griffoni E., Lecouvey M. (2020). Formation of 1-Hydroxymethylene-1,1-bisphosphinates through the Addition of a Silylated Phosphonite on Various Trivalent Derivatives. J. Org. Chem..

[11] Dussart-Gautheret J., Deschamp J., Legigan T., Monteil M., Migianu-Griffoni E., Lecouvey M. (2021). One-Pot Synthesis of Phosphinylphosphonate Derivatives and Their Anti-Tumor Evaluations. Molecules.

[12] Barbosa J.S., Almeida Paz F.A., Braga S.S. (2021). Bisphosphonates, Old Friends of Bones and New Trends in Clinics. J. Med. Chem..

[13] Dussart J., Deschamp J., Migianu-Griffoni E., Lecouvey M. (2020). From Industrial Method to the Use of Silylated P(III) Reagents for the Synthesis of Relevant Phosphonylated Molecules. Org. Process Res. Dev..

[14] Reszka A.A., Rodan G.A. (2004). Nitrogen-Containing Bisphosphonate Mechanism of Action. Mini Rev. Med. Chem..

[15] Ebetino F.H., Hogan A.M., Sun S., Tsoumpra M.K., Duan X., Triffitt J.T., Kwaasi A.A., Dunford J.E., Barnett B.L., Oppermann U. (2011). The relationship between the chemistry and biological activity of the bisphosphonates. Bone.

[16] Clézardin P. (2011). Bisphosphonates’ antitumor activity: An unravelled side of a multifaceted drug class. Bone.

[17] Rogers M.J., Frith J.C., Luckman S.P., Coxon F.P., Benford H.L., Mönkkönen J., Auriola S., Chilton K.M., Russell R.G.G. (1999). Molecular mechanisms of action of bisphosphonates. Bone.

[18] Lin J.H. (1996). Bisphosphonates: A review of their pharmacokinetic properties. Bone.

[19] Mimura M., Hayashida M., Nomiyama K., Ikegami S., Iida Y., Tamura M., Hiyama Y., Ohishi Y. (1993). Synthesis and Evaluation of (Piperidinomethylene)bis(phosphonic acid) Derivatives as Anti-osteoporosis Agents. Chem. Pharm. Bull..

[20] Szajnman S.H., Ravaschino E.L., Docampo R., Rodriguez J.B. (2005). Synthesis and biological evaluation of 1-amino-1,1-bisphosphonates derived from fatty acids against Trypanosoma cruzi targeting farnesyl pyrophosphate synthase. Bioorg. Med. Chem. Lett..

[21] Kotsikorou E., Song Y., Chan J.M.W., Faelens S., Tovian Z., Broderick E., Bakalara N., Docampo R., Oldfield E. (2005). Bisphosphonate Inhibition of the Exopolyphosphatase Activity of the Trypanosoma brucei Soluble Vacuolar Pyrophosphatase. J. Med. Chem..

[22] Leon A., Liu L., Yang Y., Hudock M.P., Hall P., Yin F., Studer D., Puan K.-J., Morita C.T., Oldfield E. (2006). Isoprenoid Biosynthesis as a Drug Target:  Bisphosphonate Inhibition of Escherichia coli K12 Growth and Synergistic Effects of Fosmidomycin. J. Med. Chem..

[23] Occhipinti A., Berlicki L., Giberti S., Dziedziola G., Kafarski P., Forlani G. (2010). Effectiveness and mode of action of phosphonate inhibitors of plant glutamine synthetase. Pest Manag. Sci..

[24] Kafarski P., Lejczak B., Forlani G. (2000). Herbicidally active aminomethylenebisphosphonic acids. Heteroat. Chem..

[25] Simoni D., Gebbia N., Invidiata F.P., Eleopra M., Marchetti P., Rondanin R., Baruchello R., Provera S., Marchioro C., Tolomeo M. (2008). Design, Synthesis, and Biological Evaluation of Novel Aminobisphosphonates Possessing an in Vivo Antitumor Activity Through a γδ-T Lymphocytes-Mediated Activation Mechanism. J. Med. Chem..

[26] Chmielewska E., Kafarski P. (2016). Synthetic Procedures Leading towards Aminobisphosphonates. Molecules.

[27] Hong Y.C., Ye J.L., Huang P.Q. (2022). One-Pot Synthesis of α-Amino Bisphosphonates from Nitriles via Tf_2_O/HC(OR)_3_-Mediated Interrupted Ritter-Type Reaction. J. Org. Chem..

[28] Kaboudin B., Esfandiari H., Moradi A., Kazemi F., Aoyama H. (2019). ZnCl_2_-Mediated Double Addition of Dialkylphosphite to Nitriles for the Synthesis of 1-Aminobisphosphonates. J. Org. Chem..

[29] Islas R.E., García J.J. (2019). Nickel-Catalyzed Hydrophosphonylation and Hydrogenation of Aromatic Nitriles Assisted by Lewis Acid. ChemCatChem.

[30] Wang A.E., Chang Z., Sun W.T., Huang P.Q. (2015). General and chemoselective bisphosphonylation of secondary and tertiary amides. Org. Lett..

[31] Prishchenko A.A., Alekseyev R.S., Livantsov M.V., Novikova O.P., Livantsova L.I., Petrosyan V.S. (2020). Synthesis of new functionalized aryl and pyridyl aminomethylenebisphosphonic acids and their derivatives via silicon-assisted methodology. J. Organomet. Chem..

[32] Prishchenko A.A., Alekseyev R.S., Livantsov M.V., Novikova O.P., Livantsova L.I., Petrosyan V.S. (2020). Silicon-assisted synthesis of new aminomethylenebisphosphonic acids with quinolines moieties. J. Organomet. Chem..

[33] Prishchenko A.A., Alekseyev R.S., Livantsov M.V., Novikova O.P., Livantsova L.I., Petrosyan V.S. (2018). Organosilicon based synthesis of new functionalized aminomethylenediphosphonates with moieties of amino acids. J. Organomet. Chem..

[34] Prishchenko A.A., Alekseyev R.S., Livantsov M.V., Novikova O.P., Livantsova L.I., Petrosyan V.S. (2018). Tris(trimethylsilyl) phosphite as key synthon for convenient synthesis of new organosilicon(phosphorus)-containing N-heterocycles. J. Organomet. Chem..

[35] Prishchenko A.A., Alekseyev R.S., Livantsov M.V., Novikova O.P., Livantsova L.I., Terenin V.I., Petrosyan V.S. (2017). Synthesis of new functionalized mono- and diphosphonic acids with five-membered aza-heterocycles moieties. Heteroat. Chem..

[36] Prishchenko A.A., Livantsov M.V., Novikova O.P., Livantsova L.I., Ershov I.S., Petrosyan V.S. (2015). Synthesis of new types of aminomethylenediphosphorus-containing acids and their derivatives. Russ. J. Gen. Chem..

[37] Prishchenko A.A., Livantsov M.V., Novikova O.P., Livantsova L.I., Ershov I.S., Petrosyan V.S. (2015). Synthesis of the New Types of *N*-Unsubstituted Aminomethylenebisorganophosphorus Acids and Their Derivatives. Heteroat. Chem..

[38] Minaeva L.I., Patrikeeva L.S., Kabachnik M.M., Beletskaya I.P., Orlinson B.S., Novakov I.A. (2011). Synthesis of novel aminomethylenebisphosphonates and bisphosphonic acids, containing adamantyl fragment. Heteroat. Chem..

[39] Dąbrowska E., Burzyńska A., Mucha A., Matczak-Jon E., Sawka-Dobrowolska W., Berlicki Ł., Kafarski P. (2009). Insight into the mechanism of three component condensation leading to aminomethylenebisphosphonates. J. Organomet. Chem..

[40] Kaboudin B., Alipour S. (2009). A microwave-assisted solvent- and catalyst-free synthesis of aminomethylene bisphosphonates. Tetrahedron Lett..

[41] Wu M., Chen R., Huang Y. (2004). Simple, Efficient and One-Pot Method for Synthesis of Aminomethylene gem-Diphosphonic Acid Derivatives from Ketones via Beckmann Rearrangement. Synthesis.

[42] Yokomatsu T., Yoshida Y., Nakabayashi N., Shibuya S. (1994). Simple and Efficient Method for Preparation of Conformationally Constrained Aminomethylene gem-Diphosphonate Derivatives via Beckmann Rearrangement. J. Org. Chem..

[43] David T., Procházková S., Kotek J., Kubíček V., Hermann P., Lukeš I. (2014). Aminoalkyl-1,1-bis(phosphinic acids): Stability, Acid-Base, and Coordination Properties. Eur. J. Inorg. Chem..

[44] Prishchenko A.A., Livantsov M.V., Novikova O.P., Livantsova L.I., Ershov I.S., Petrosyan V.S. (2013). Synthesis and Reactivity of the New Trimethylsilyl Esters of Aminomethylenebisorganophosphorus Acids. Heteroat. Chem..

[45] Prishchenko A.A., Livantsov M.V., Novikova O.P., Livantsova L.I., Ershov I.S., Petrosyan V.S. (2013). Synthesis of N-substituted aminomethylenediphosphonites and their derivatives. Russ. J. Gen. Chem..

[46] Samples M.S., Yoder C.H. (1987). The structure of bis(organosily1) amides containing the dimethylsilyl and bis(dimethylsilyl) ethylene groups. J. Organomet. Chem..

[47] Klebe J.F., Finkbeiner H., White D.M. (1966). Silylations with Bis(trimethylsilyl)acetamide, a Highly Reactive Silyl Donor. J. Am. Chem. Soc..

[48] Knapp S.B.S., Gibson F.S. (1992). IODOLACTAMIZATION: 8-exo-iodo-2-azabicyclo[3.3.0]octan-3-one. Org. Synth..

[49] Knapp S., Levorse A.T. (1988). Synthesis and reactions of iodo lactams. J. Org. Chem..

[50] Hanada S., Motoyama Y., Nagashima H. (2008). Hydrosilanes Are Not Always Reducing Agents for Carbonyl Compounds but Can Also Induce Dehydration: A Ruthenium-Catalyzed Conversion of Primary Amides to Nitriles. Eur. J. Org. Chem..

[51] Dwak B., Lasocki Z. (1983). Structure and tautomerism of cyclic silylamides I. Disiloxane derivatives of acetamide and benzamides. J. Organomet. Chem..

[52] Bassindale A.R., Posner T.B. (1979). The Structure of Silylated Amides-. N-Methyl-NTrimethylsilyltrifluoroacetamide, a Reassignment of Structure. J. Organomet. Chem..

[53] Itoh K., Katsuda M., Ishii Y. (1970). Reactions of Group IV Organometallic Compounds. Part X1X.l Substituent Effects on the Rate of Trimethylsilyl Migration in Substituted N,O-Bis(trimethylsilyl)benzimidates. J. Chem. Soc. B.

[54] Deprèle S., Montchamp J.L. (2001). Triethylborane-Initiated Room Temperature Radical Addition of Hypophosphites to Olefins: Synthesis of Monosubstituted Phosphinic Acids and Esters. J. Org. Chem..

