# Supplementary material for: Synthesis of Aminobisphosphinates through a Cascade Reaction between Hypophosphorous Acid and Bis(trimethylsilyl)imidates Mediated by ZnI2
Source: Molecules. 2023 Aug 24;28(17):6226. doi: 10.3390/molecules28176226 (PMC10489009; doi:10.3390/molecules28176226)
Supplement: Supplementary file 1 [file molecules-28-06226-s001.zip › molecules-2563591-supplementary.pdf]

Synthesis of Aminobisphosphinates through a Cascade Reaction between Hypophosphorous Acid and Bis(trimethylsilyl)imidates Mediated by ZnI_2

.Supporting Information

1) NMR monitoring of imidate formation 2b 2
2) NMR data of 1-aminomethylene-1,1-bis(H-phosphinate) disodium salts 3a-l………3

1) NMR monitoring of imidate formation 2b

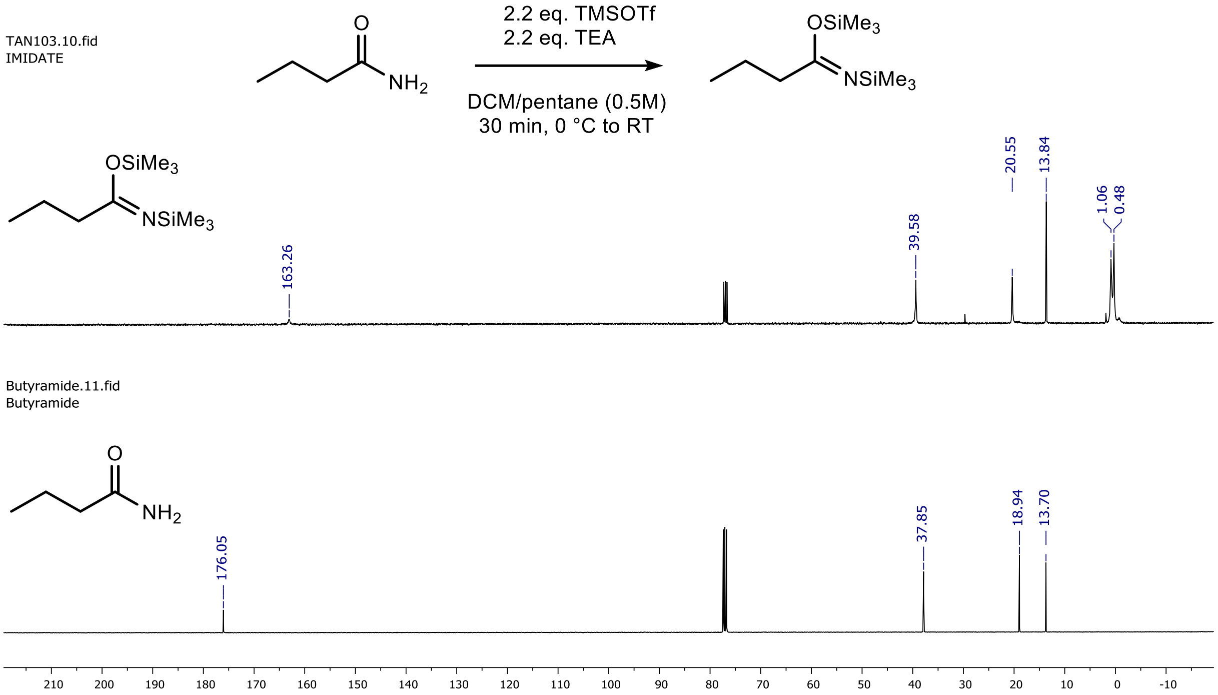

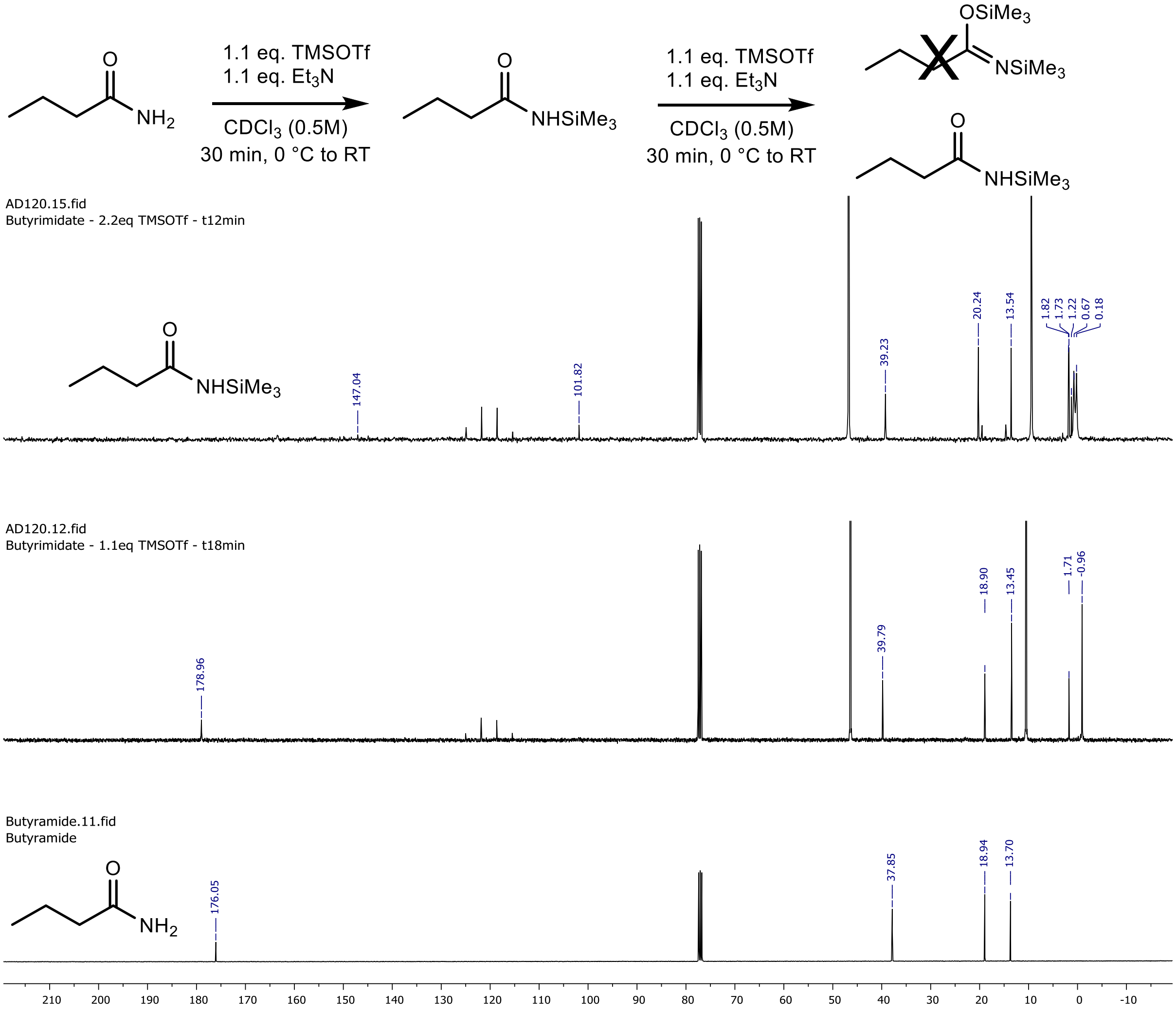

2) NMR data of 1-aminomethylene-1,1-bis(H-phosphinate) disodium salts 3a-l

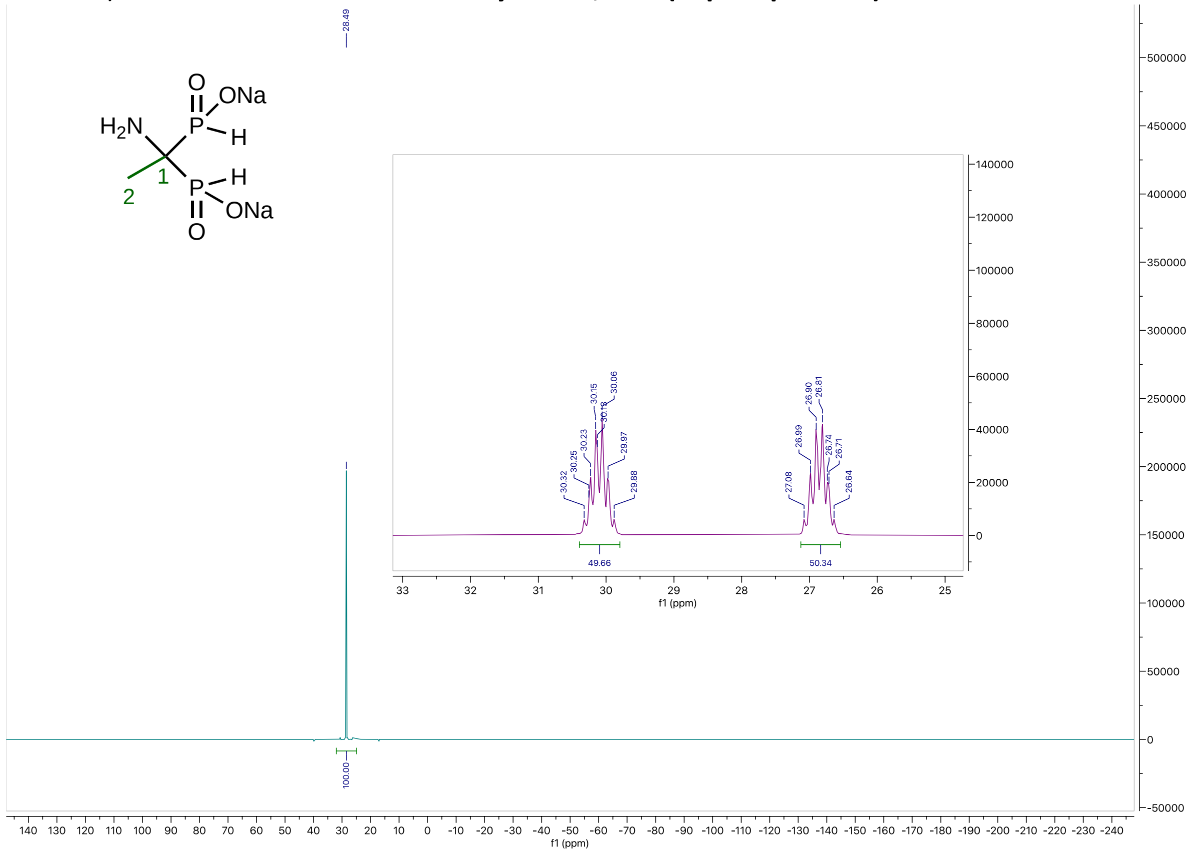

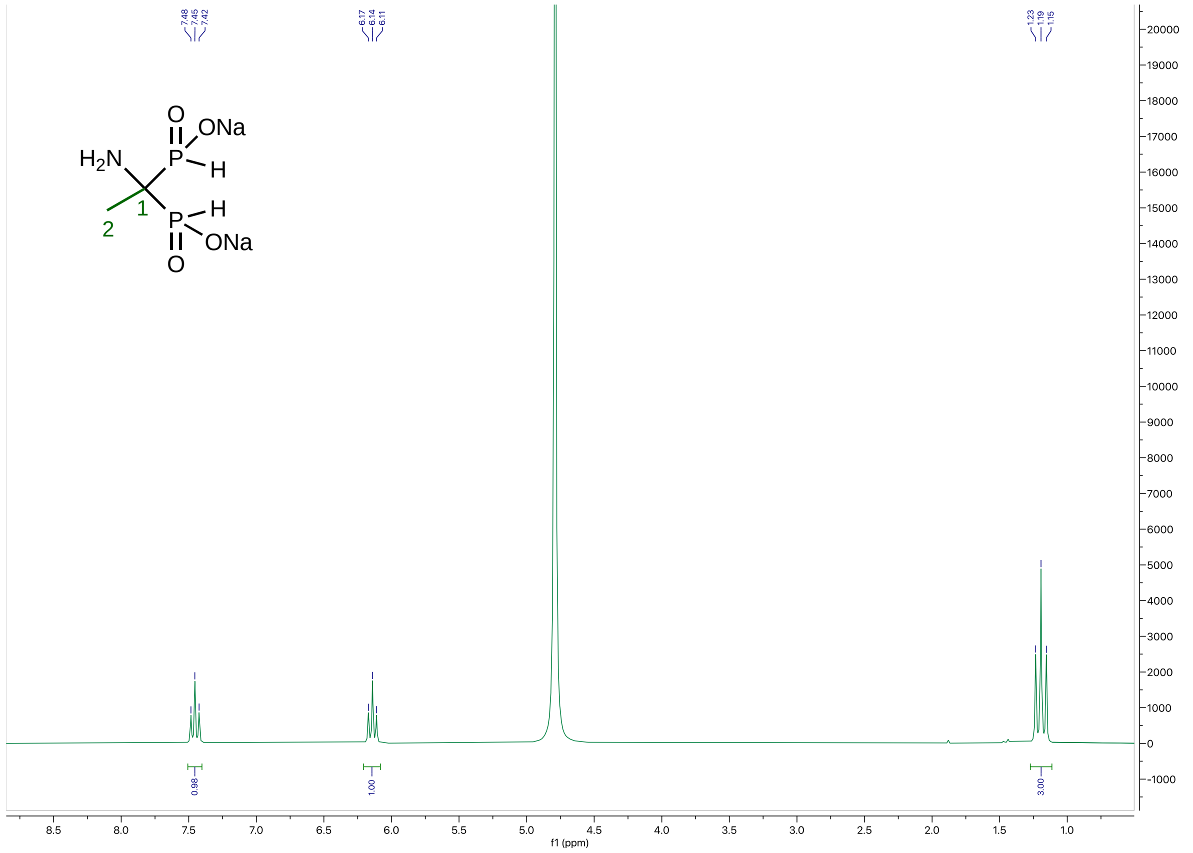

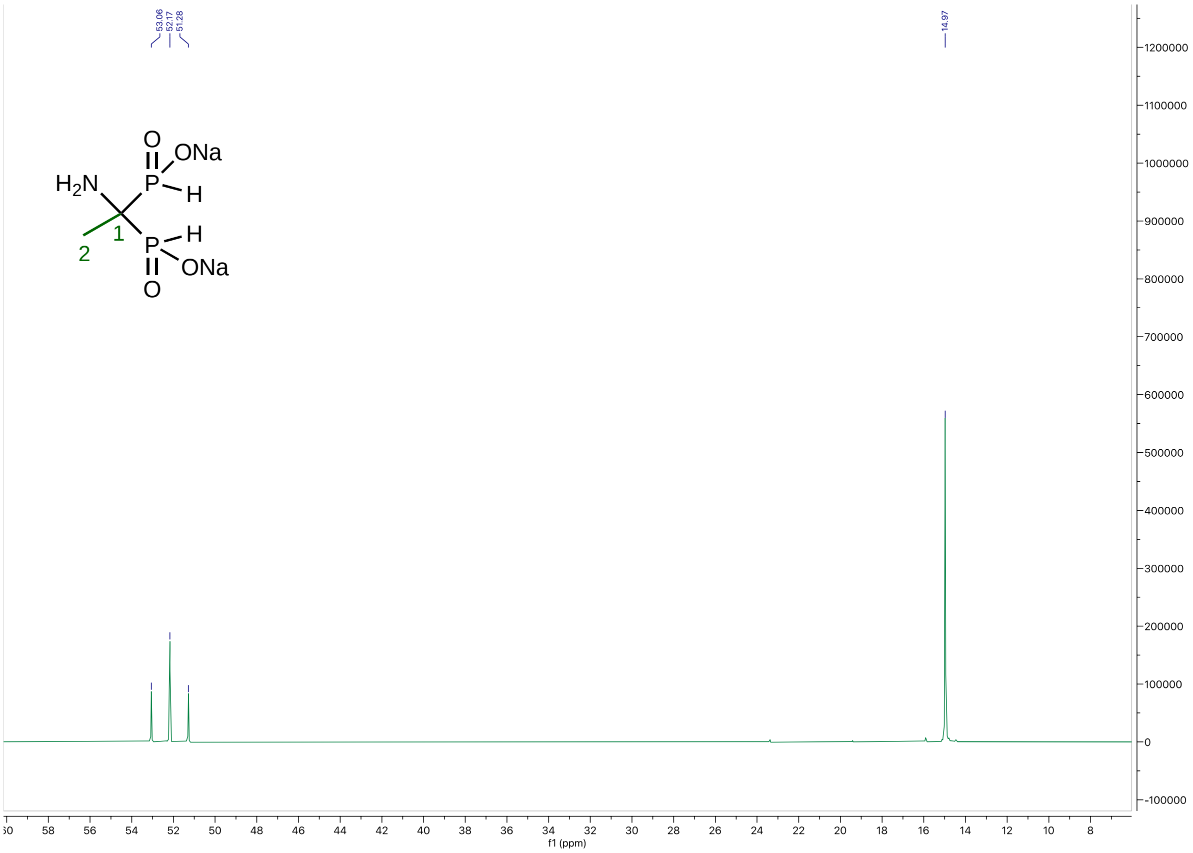

Figure S1. ^{31}P NMR spectrum (162 MHz, D_2O), ^{1}H NMR spectrum (400MHz, D_2O), ^{13}C NMR (101 MHz, D_2O) of 1-aminoethane-1,1-bis(H-phosphinate) disodium salts **3a**.

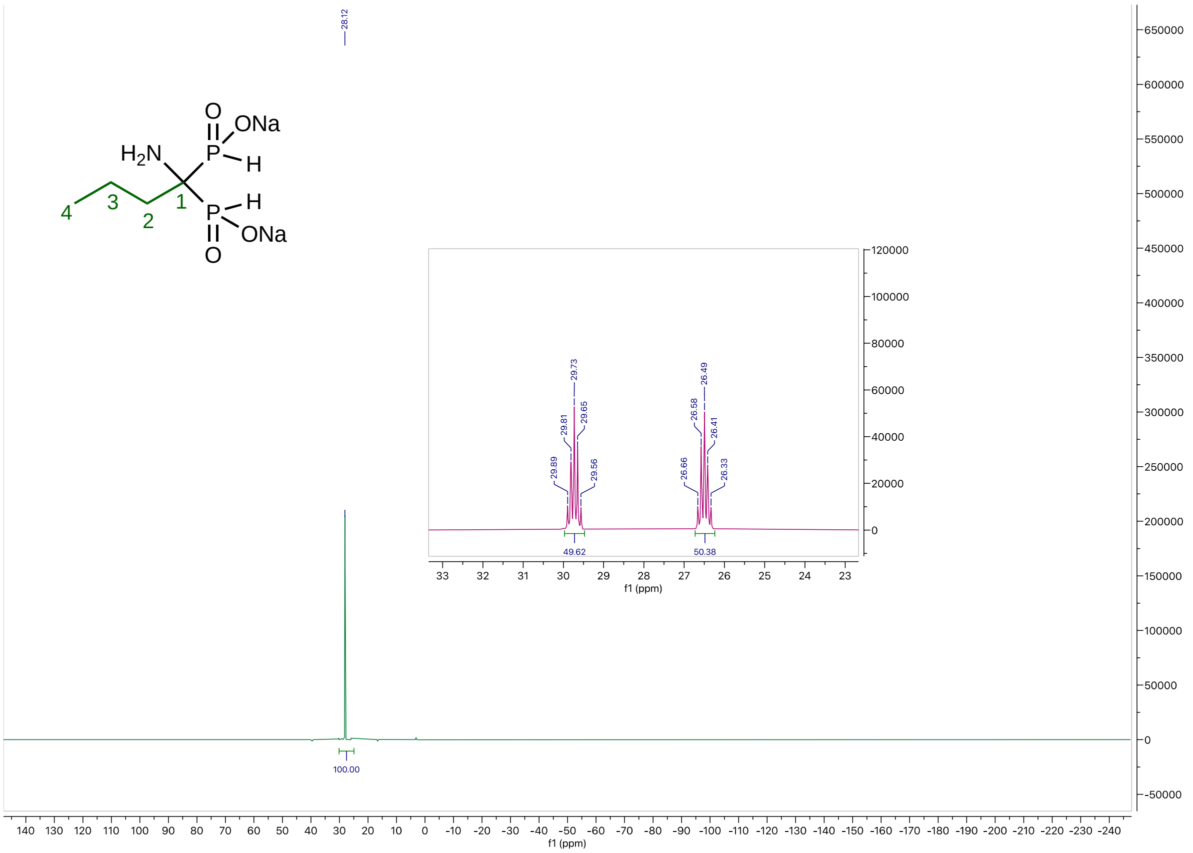

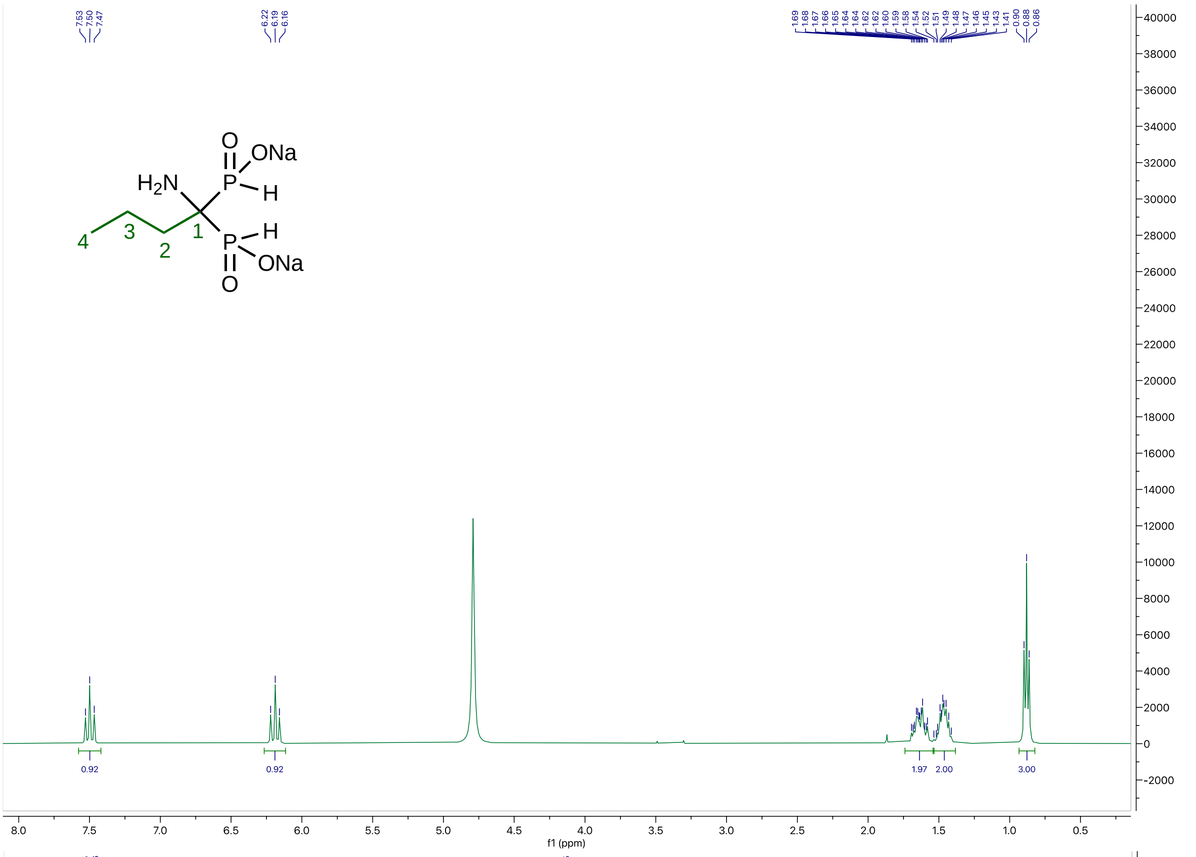

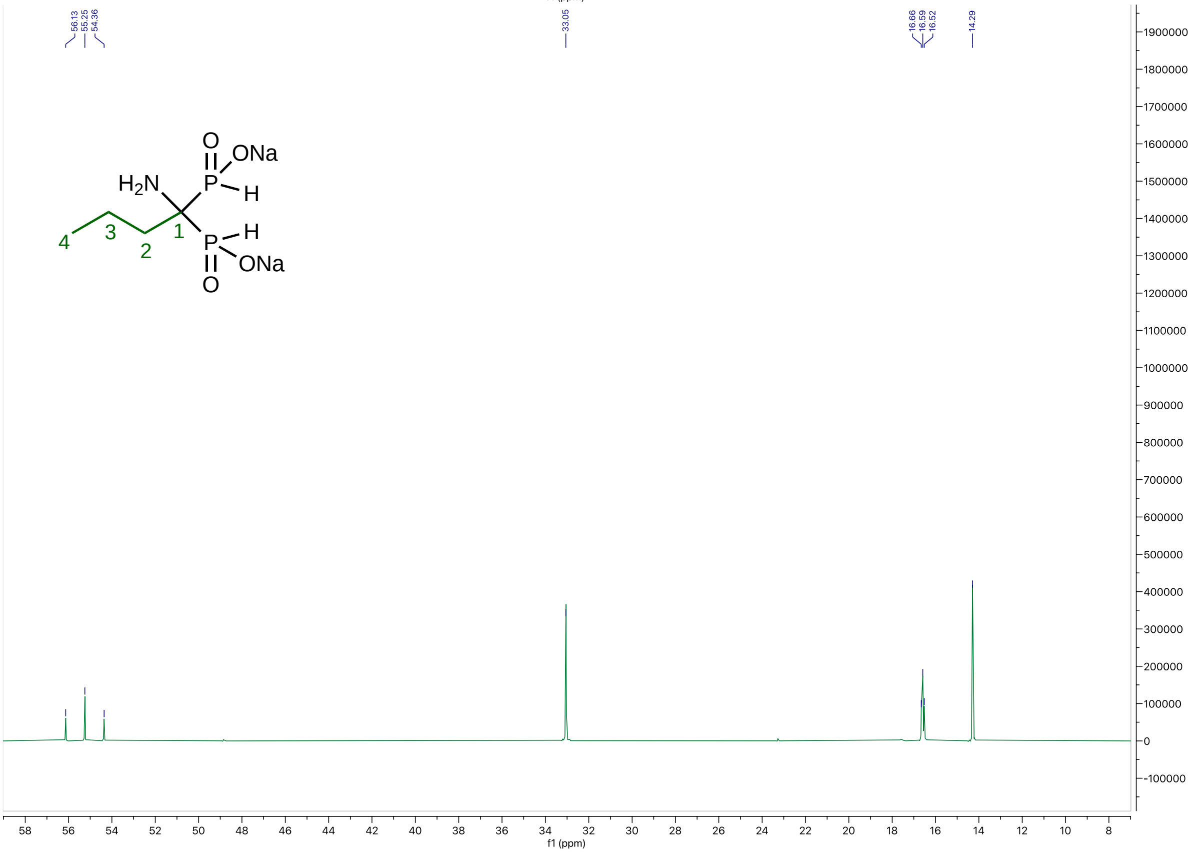

Figure S2. ^{31}P NMR spectrum (162 MHz, D_2O), ^{1}H NMR spectrum (400 MHz, D_2O), ^{13}C NMR (101 MHz, D_2O) of 1-amino-1-propylmethane-1,1-bis(H-phosphinate) disodium salts **3b**.

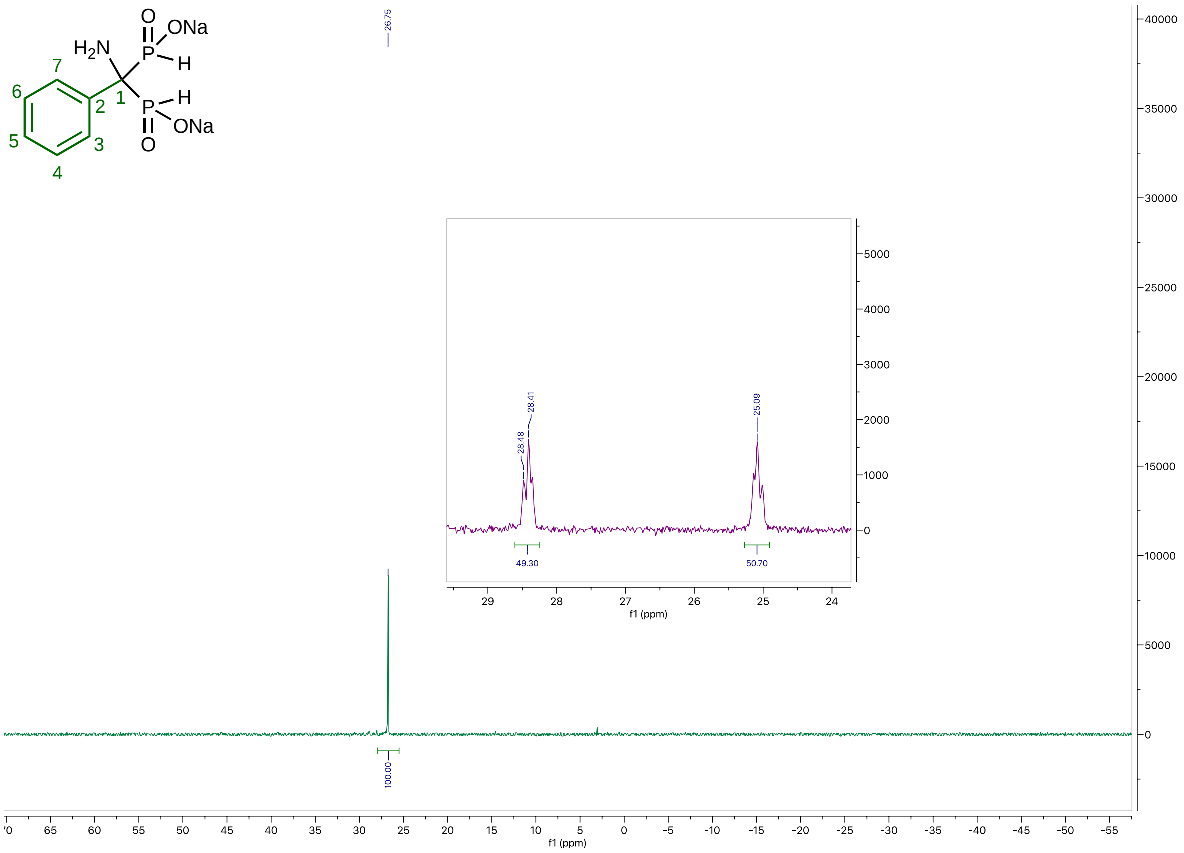

H2N
ONa
H
ONa
O
O
P
P
1
2
3
4
5
6
7
26.75
28.48
28.41
25.09
49.30
50.70
100.00
f1 (ppm)

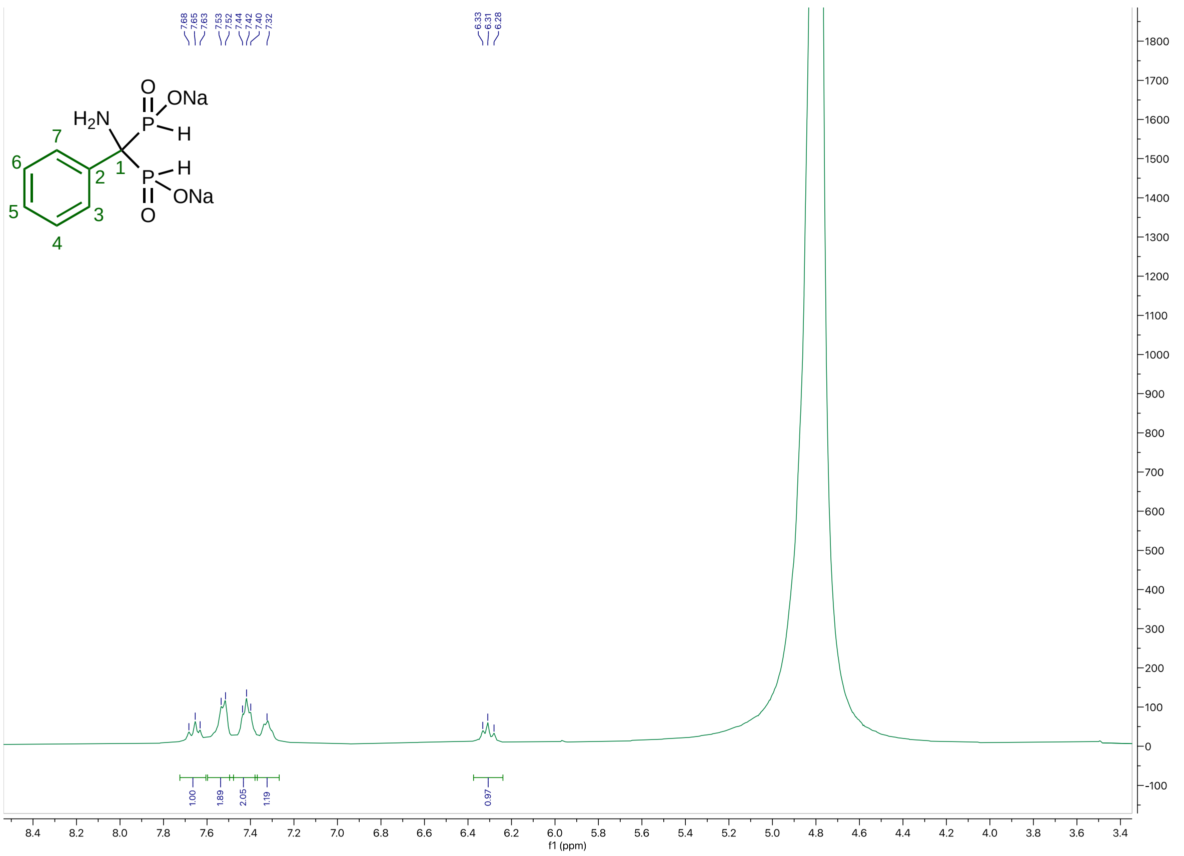

7.68
7.65
7.63
7.53
7.52
7.44
7.42
7.40
7.32
6.33
6.31
6.28
1.00
1.89
2.05
1.19
0.97
f1 (ppm)

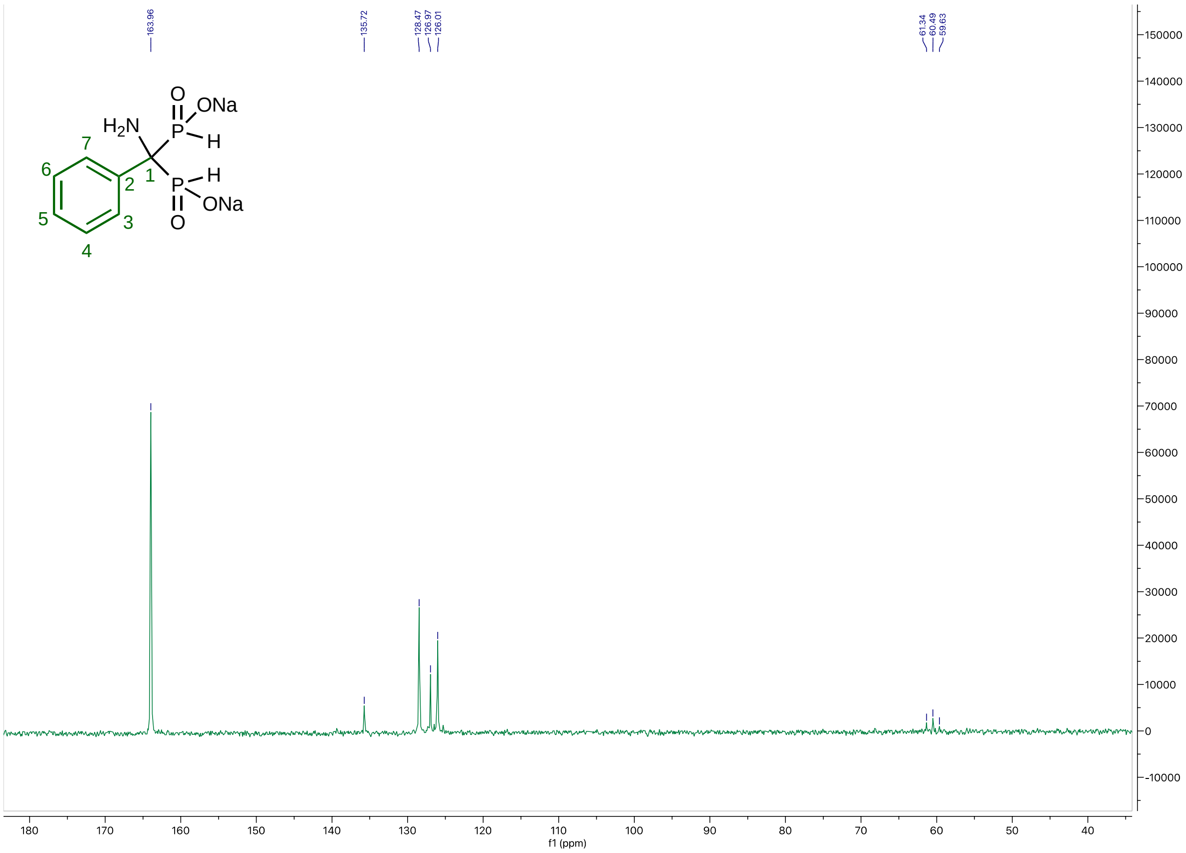

<u>Figure S3</u>. ^{31}P NMR spectrum (162 MHz, D_2O), ^{1}H NMR spectrum (400 MHz, D_2O), ^{13}C NMR (101 MHz, D_2O) of 1-amino-1-phenylmethane-1,1-bis(H-phosphinate) disodium salts **3c**.

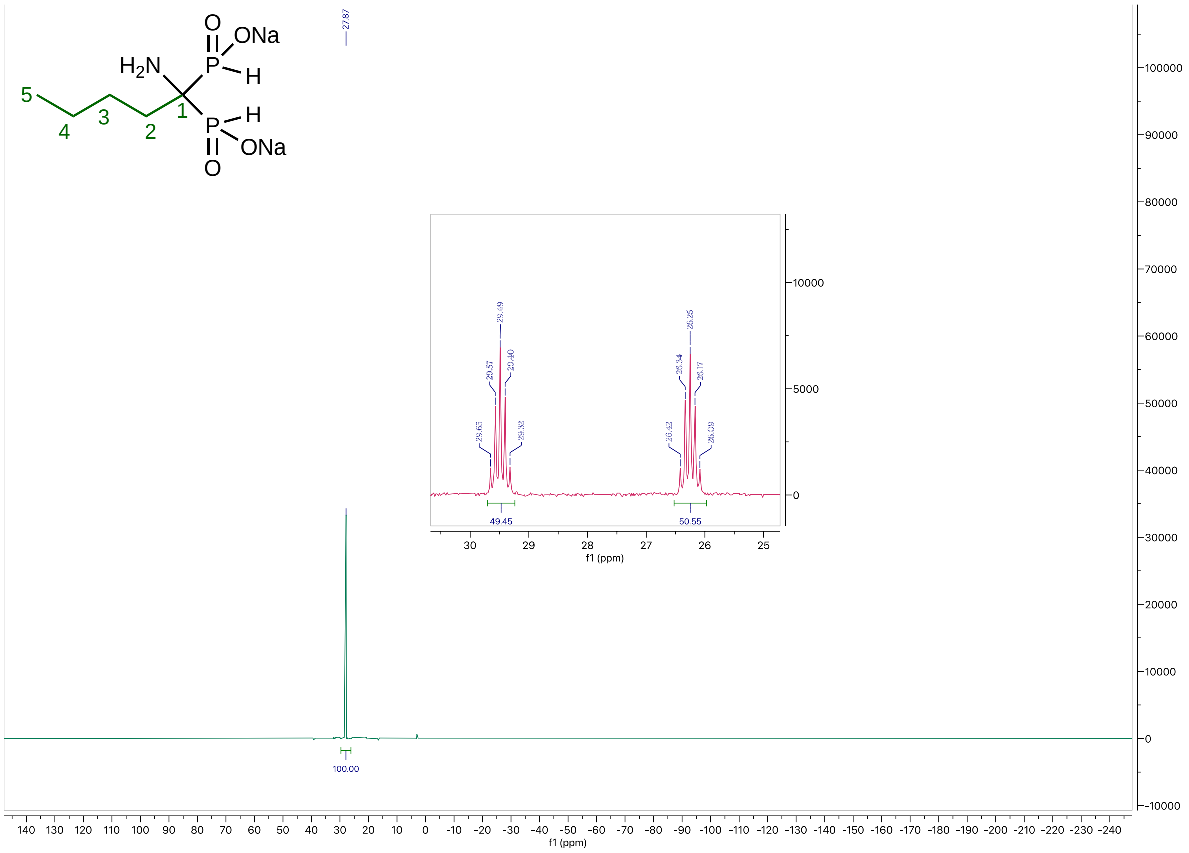

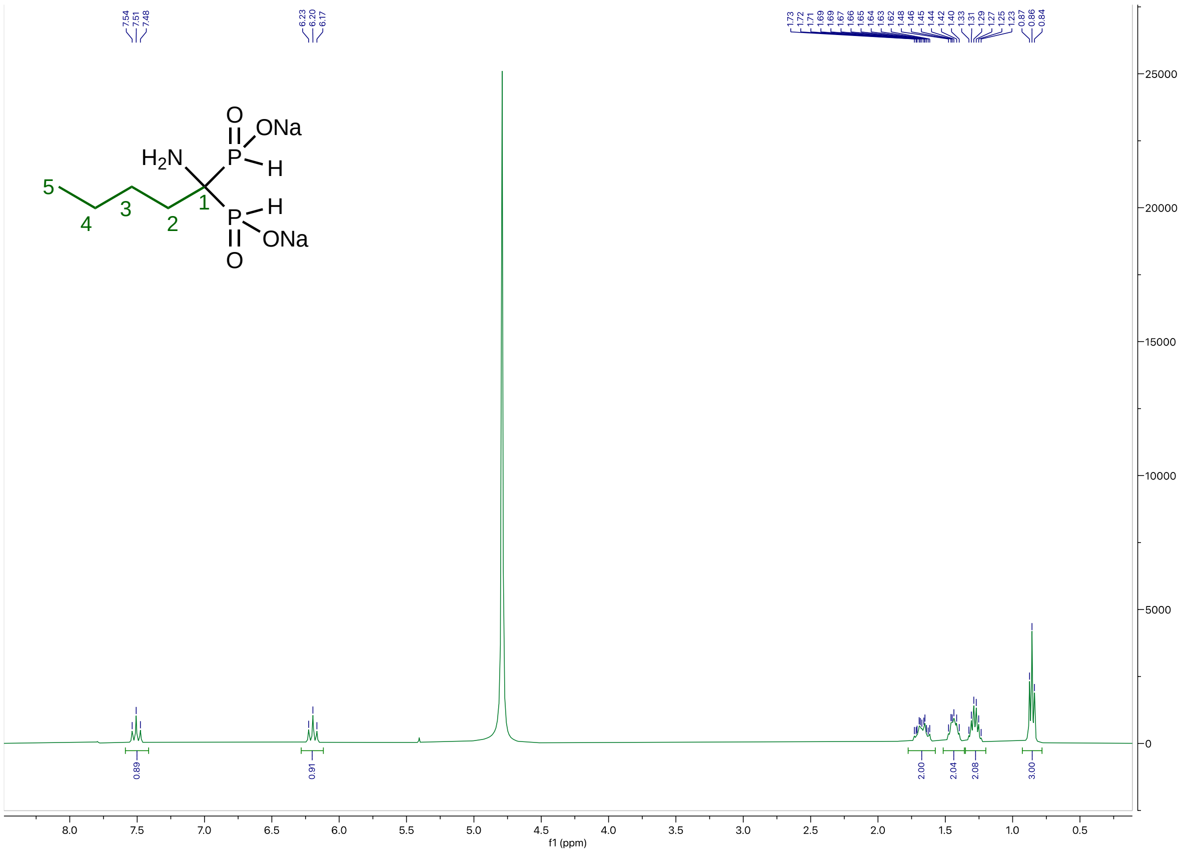

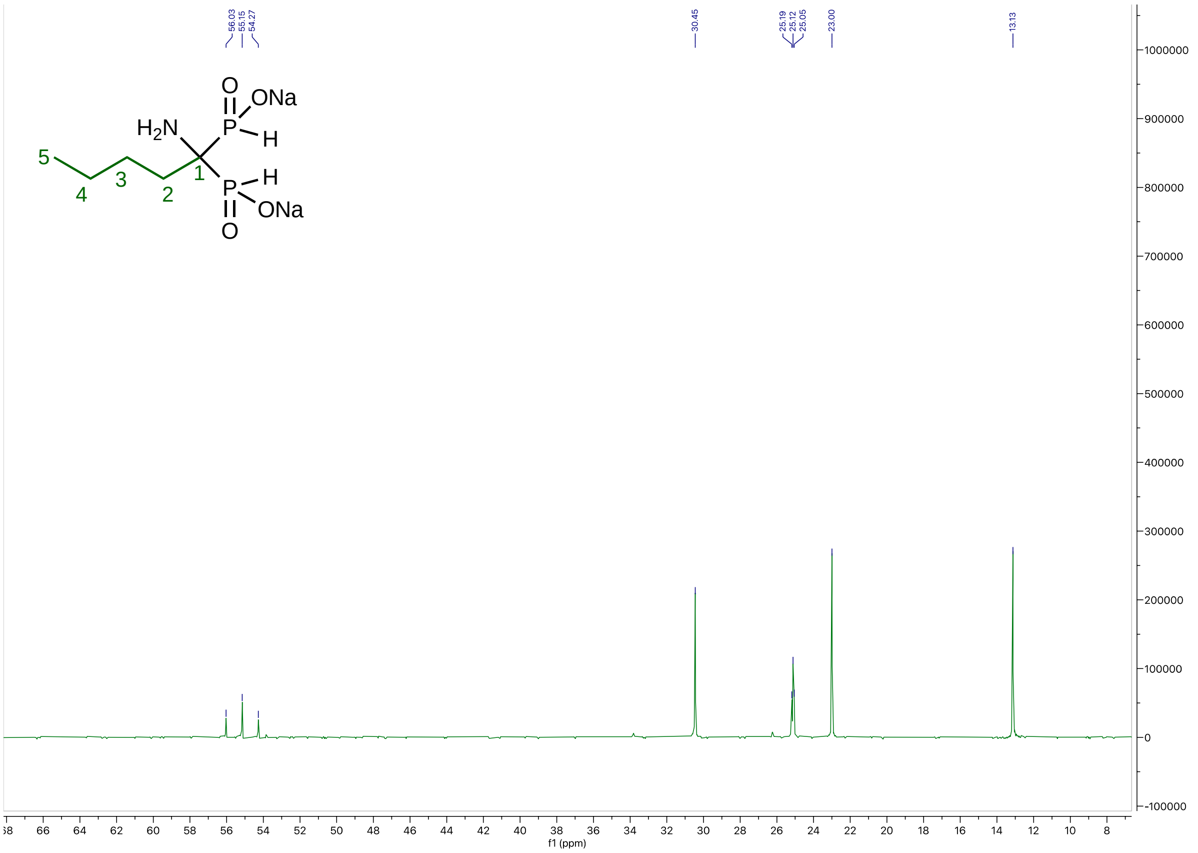

Figure S4. ^{31}P NMR spectrum (162 MHz, D_2O), ^{1}H NMR spectrum (400 MHz, D_2O), ^{13}C NMR (101 MHz, D_2O) of 1-amino-1-butylmethane-1,1-bis(H-phosphinate) disodium salts **3d**.

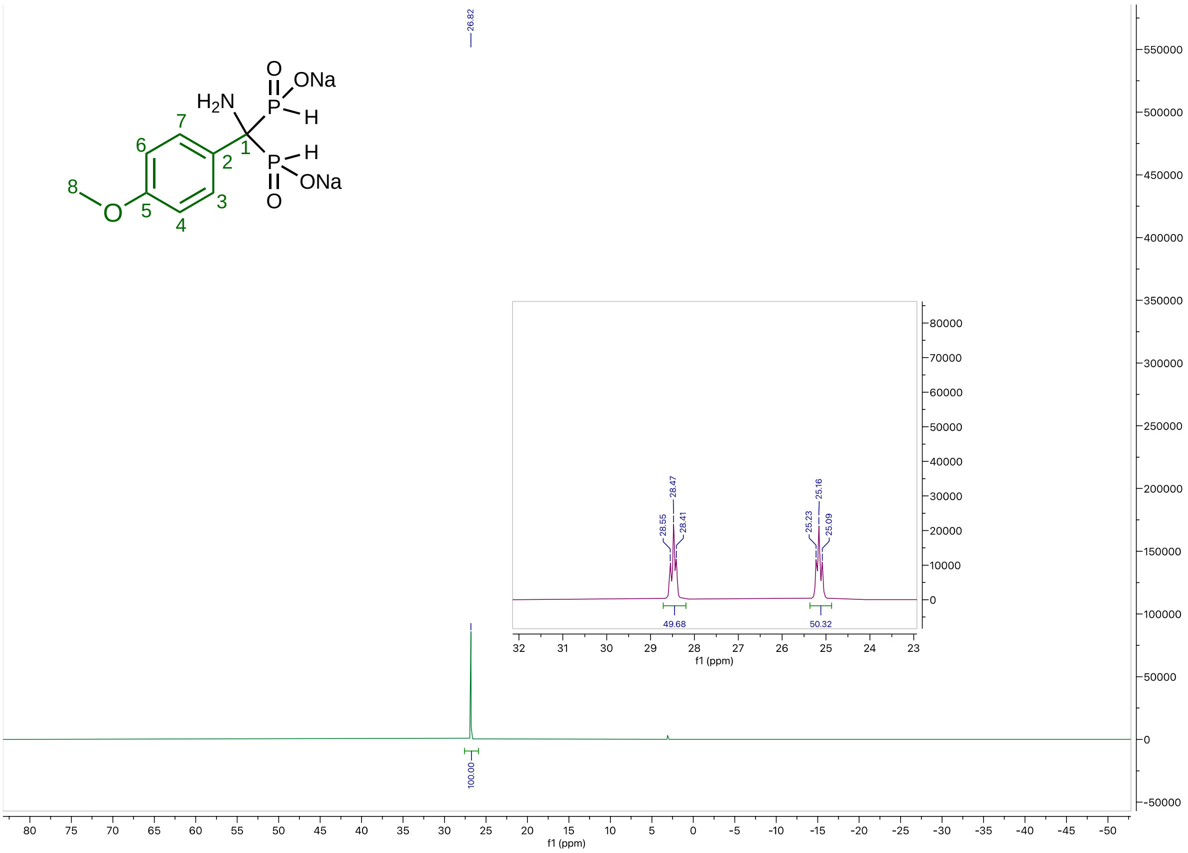

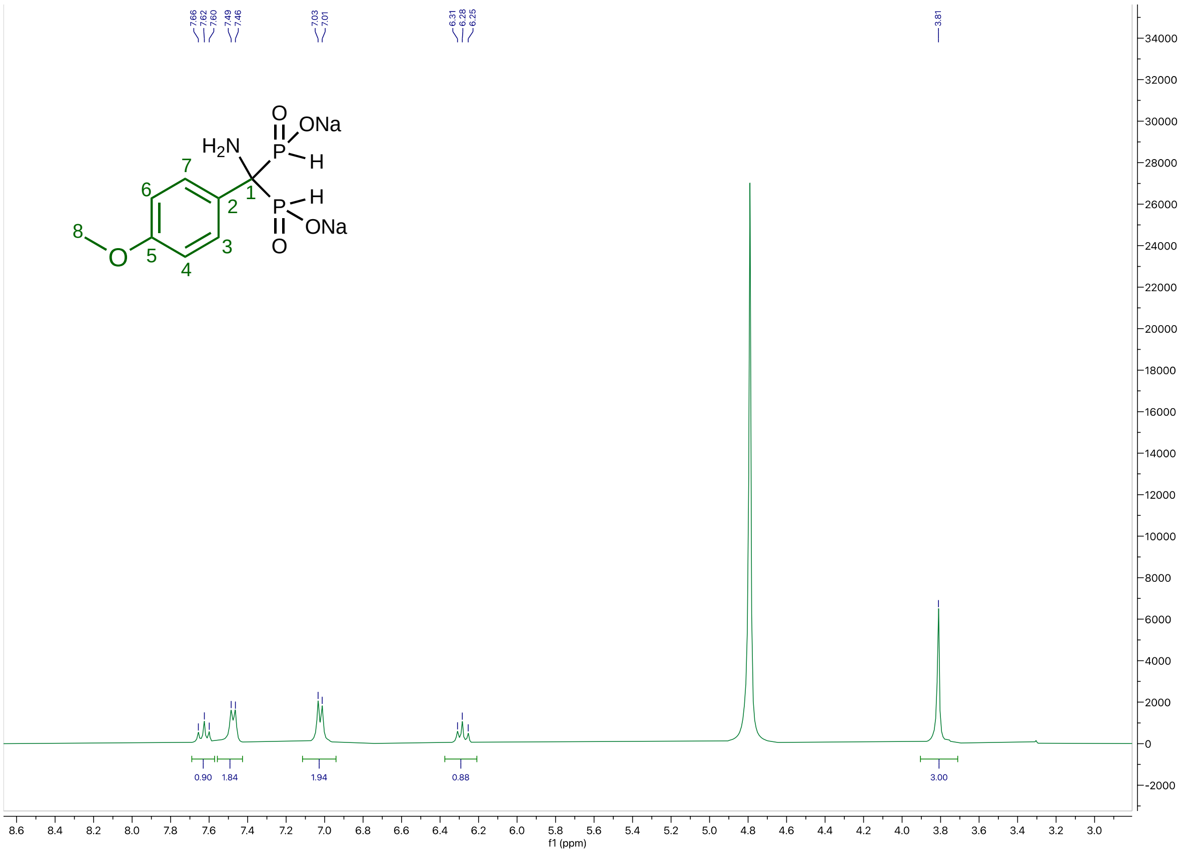

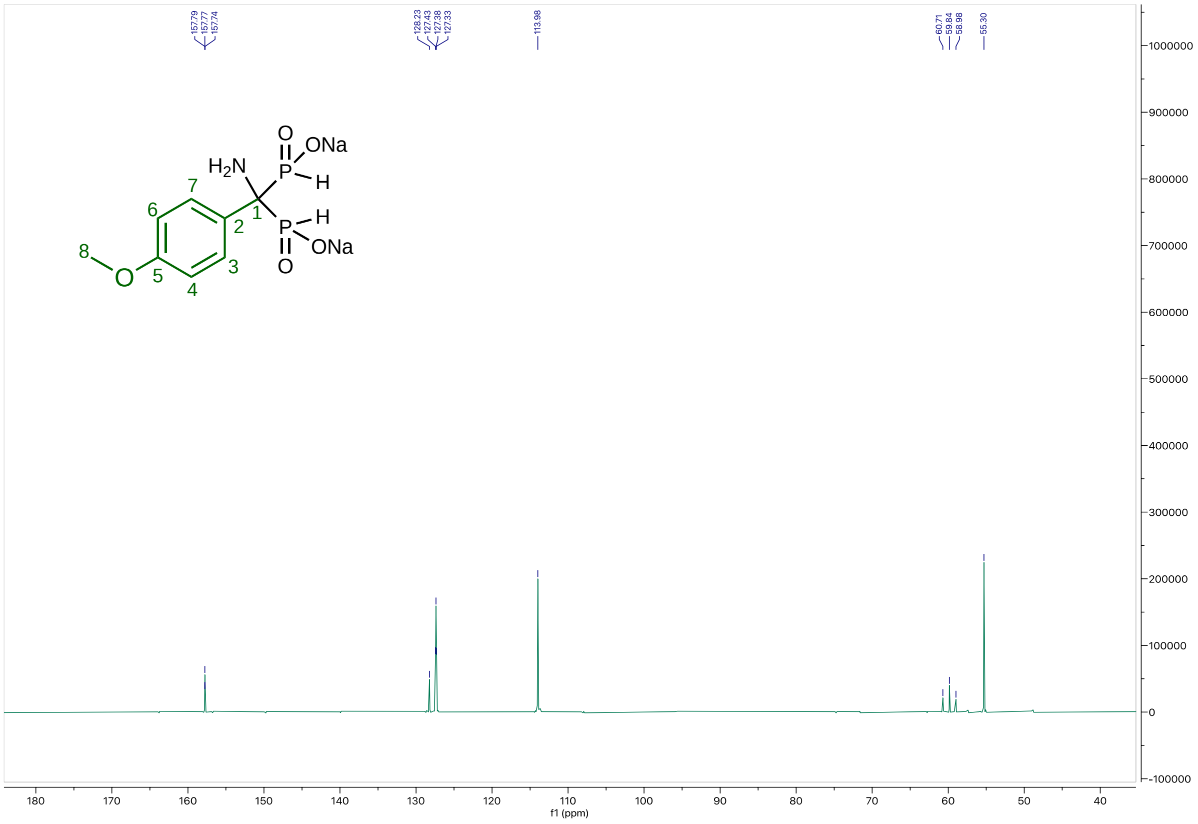

<u>Figure S5</u>. ^{31}P NMR spectrum (162 MHz, D_2O), ^{1}H NMR spectrum (400 MHz, D_2O), ^{13}C NMR (101 MHz, D_2O) of 1-amino-1-(4-methoxyphenyl)methane-1,1-bis(H-phosphinate) disodium salts **3f**.

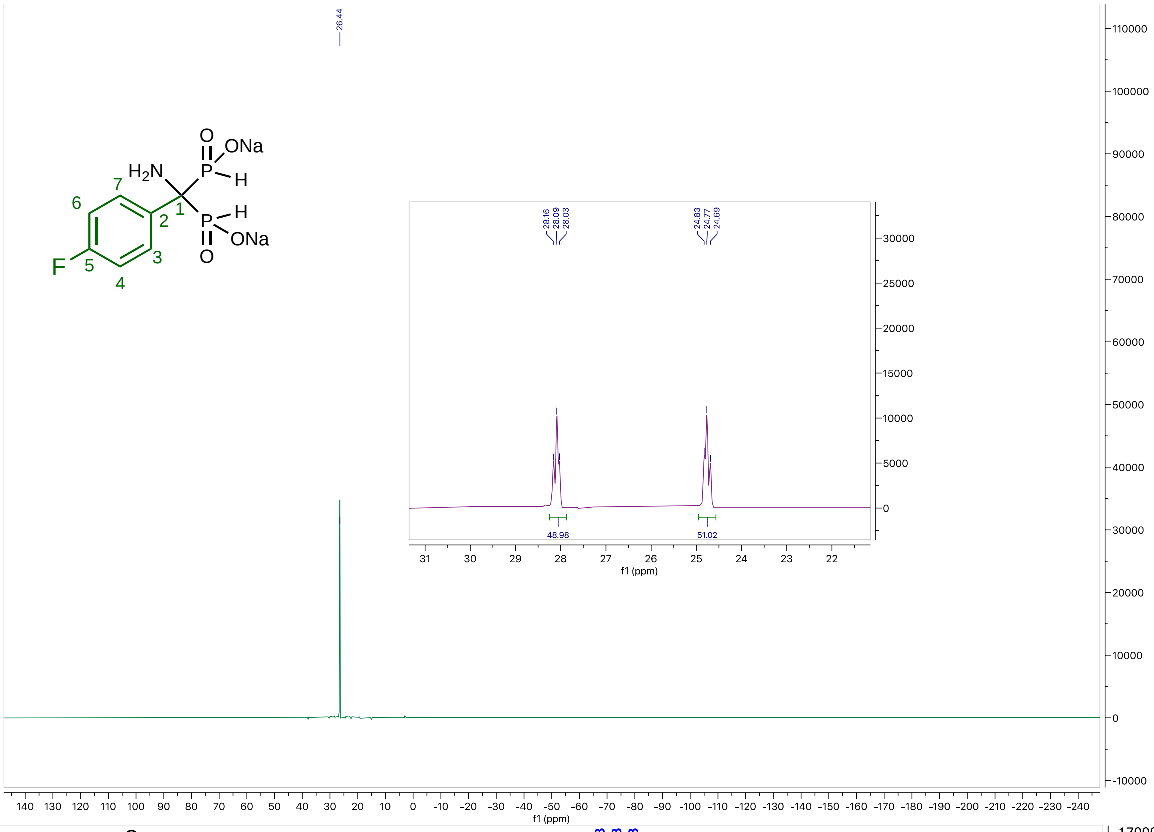
26.44
ONa
H2N
H
ONa
F
28.16
28.09
28.03
24.83
24.77
24.69
48.98
51.02
f1 (ppm)
f1 (ppm)

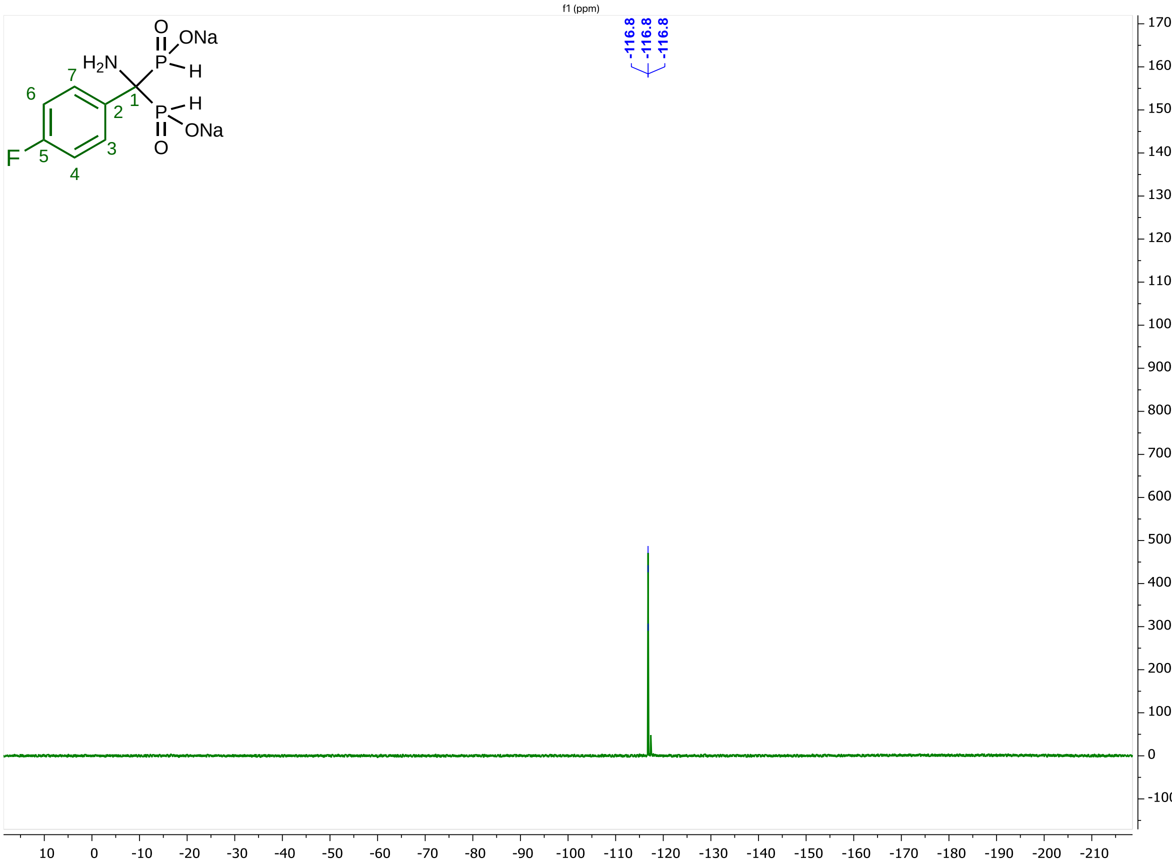
-116.8
-116.8
-116.8
ONa
H2N
H
ONa
F

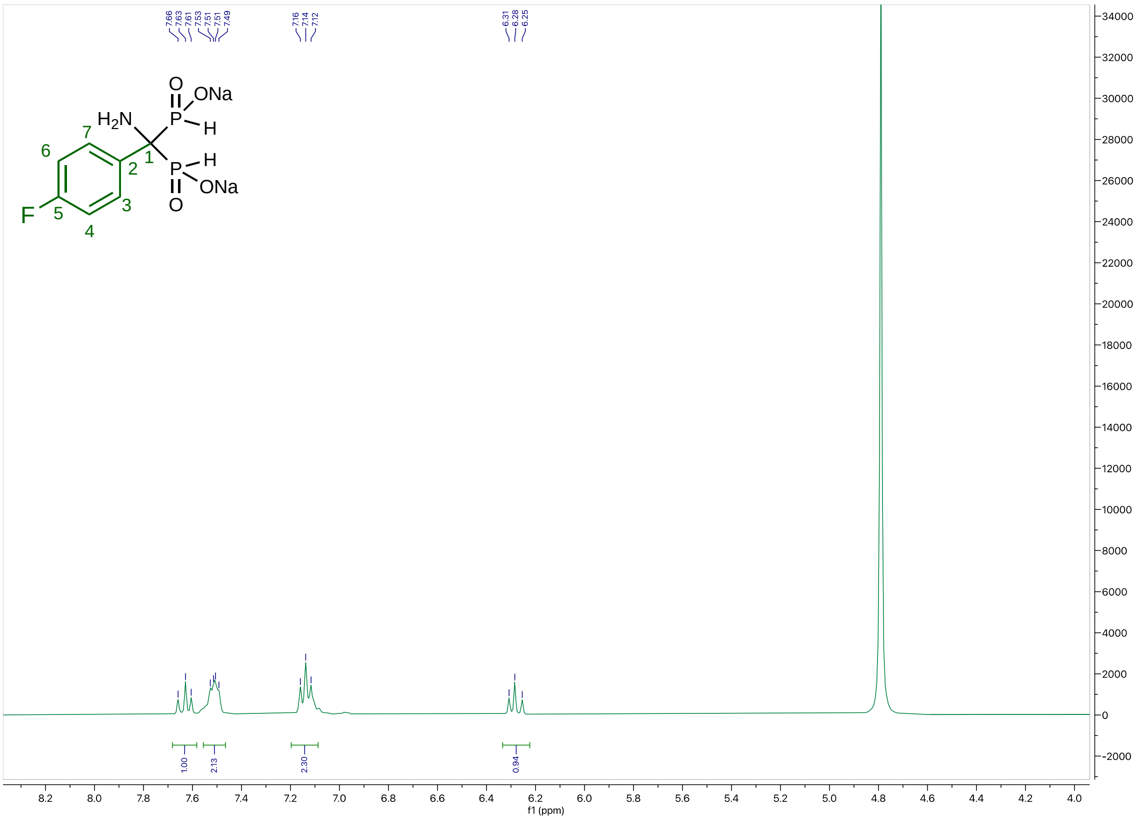

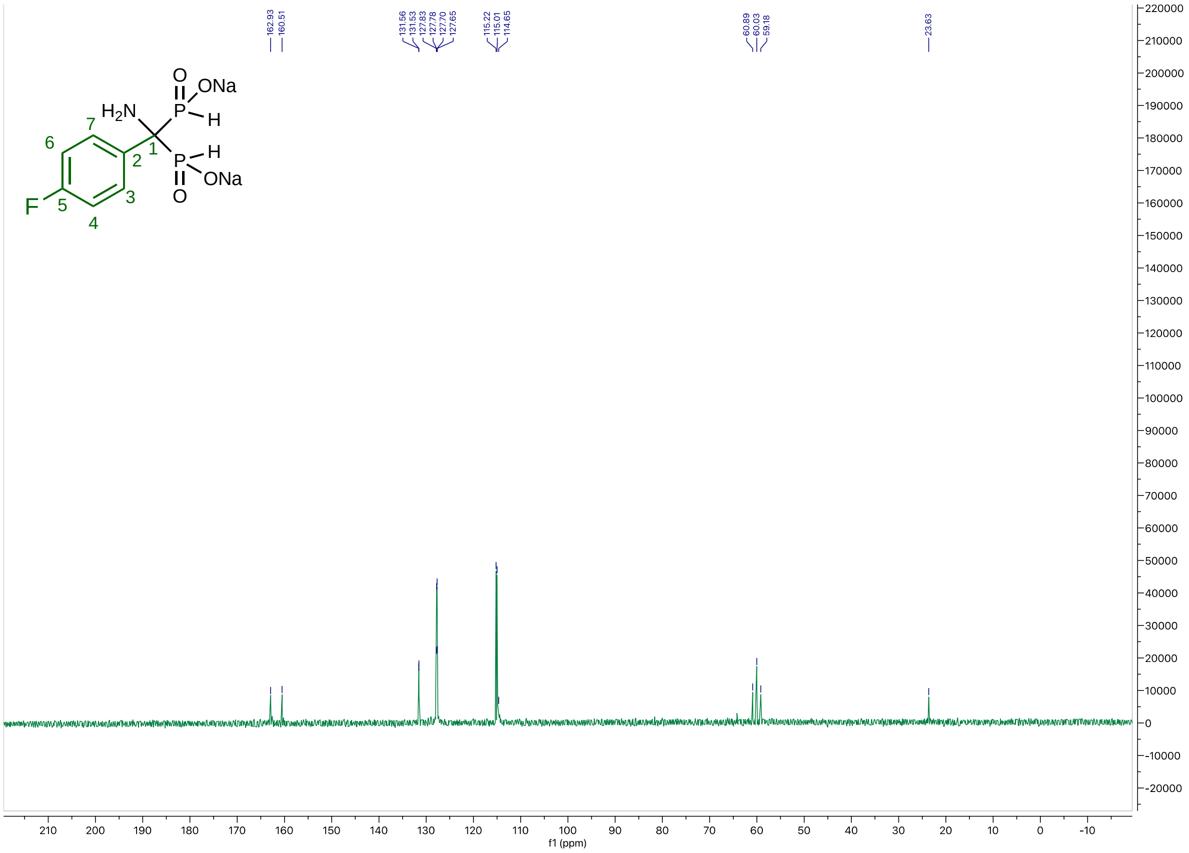

Figure S6. ^{31}P NMR spectrum (162 MHz, D_2O), ^{19}F NMR spectrum (377 MHz, D_2O), ^{1}H NMR spectrum (400MHz, D_2O), ^{13}C NMR (101 MHz, D_2O) of 1-amino-1-(4-fluorophenyl)methane-1,1-bis(H-phosphinate) disodium salts **3g**

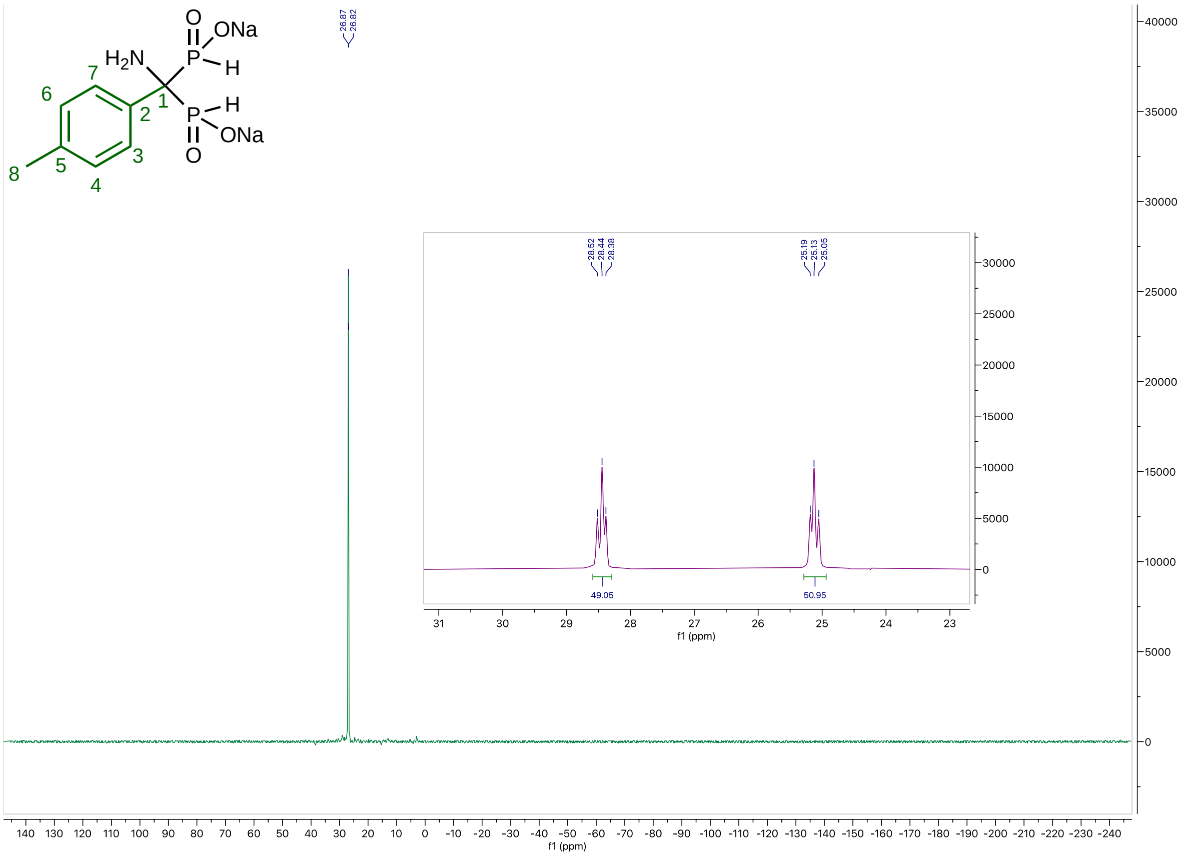
ONa
H2N
P
H
ONa
O
1
2
3
4
5
6
7
8
26.87
26.82
28.52
28.44
28.38
25.19
25.13
25.05
49.05
50.95
f1 (ppm)
f1 (ppm)

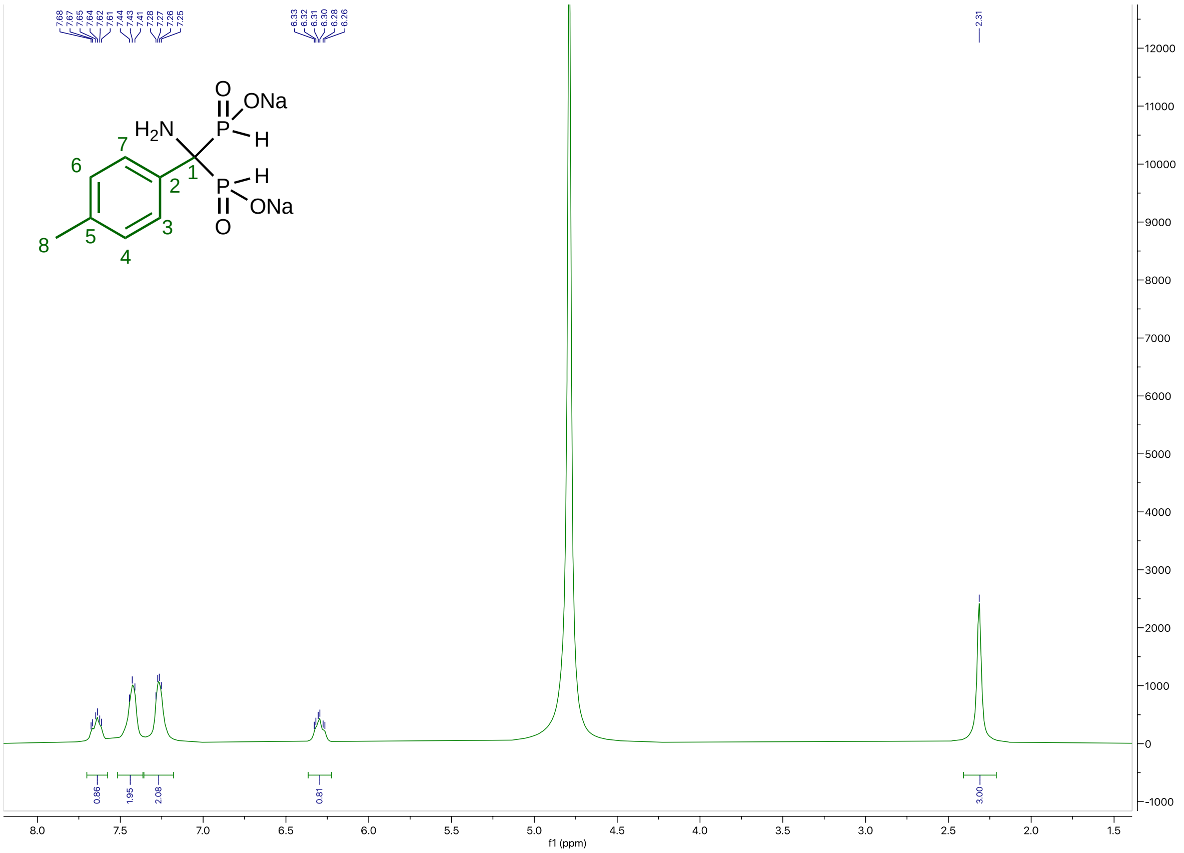
7.68
7.67
7.65
7.64
7.62
7.61
7.44
7.43
7.41
7.28
7.27
7.26
7.25
6.33
6.32
6.31
6.30
6.28
6.26
2.31
ONa
H2N
P
H
ONa
O
1
2
3
4
5
6
7
8
0.86
1.95
2.08
0.81
3.00
f1 (ppm)

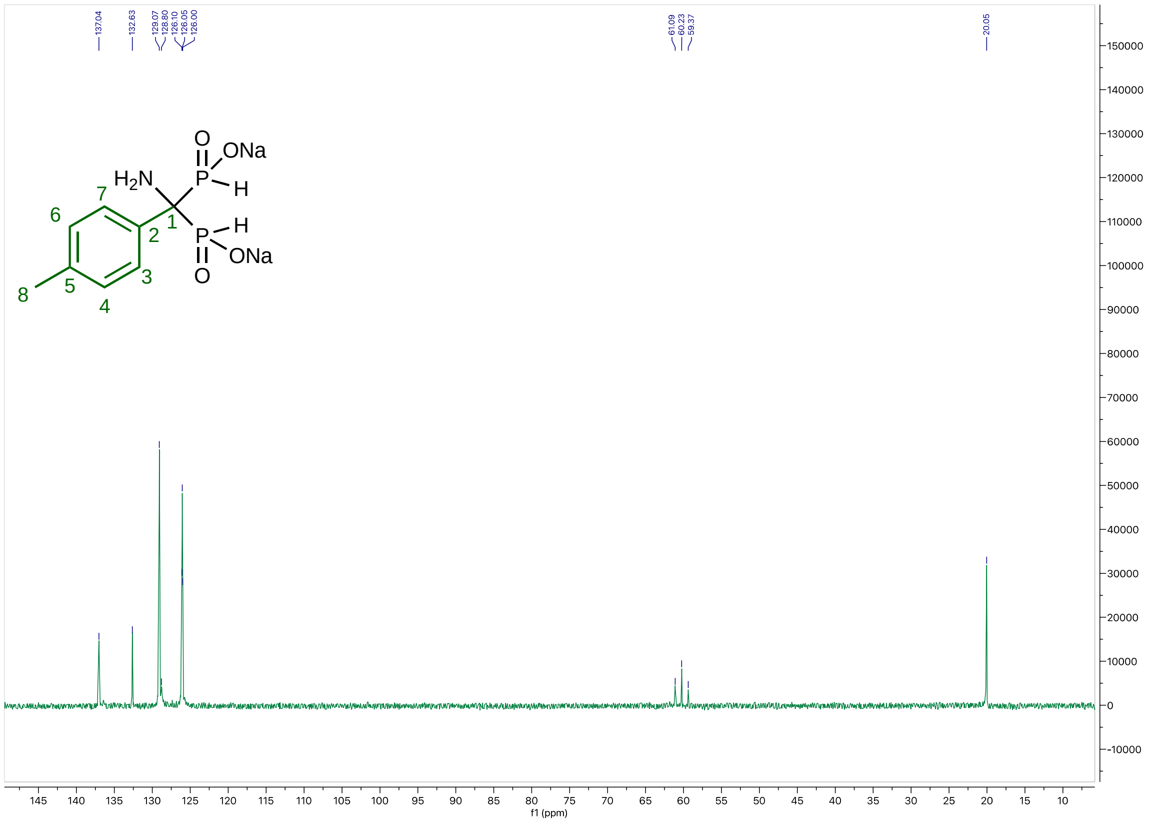

Figure S7. ^{31}P NMR spectrum (162 MHz, D_2O), ^{1}H NMR spectrum (400 MHz, D_2O), ^{13}C NMR (101 MHz, D_2O) of 1-amino-1-(4-tolyl)methane-1,1-bis(H-phosphinate) disodium salts **3i**

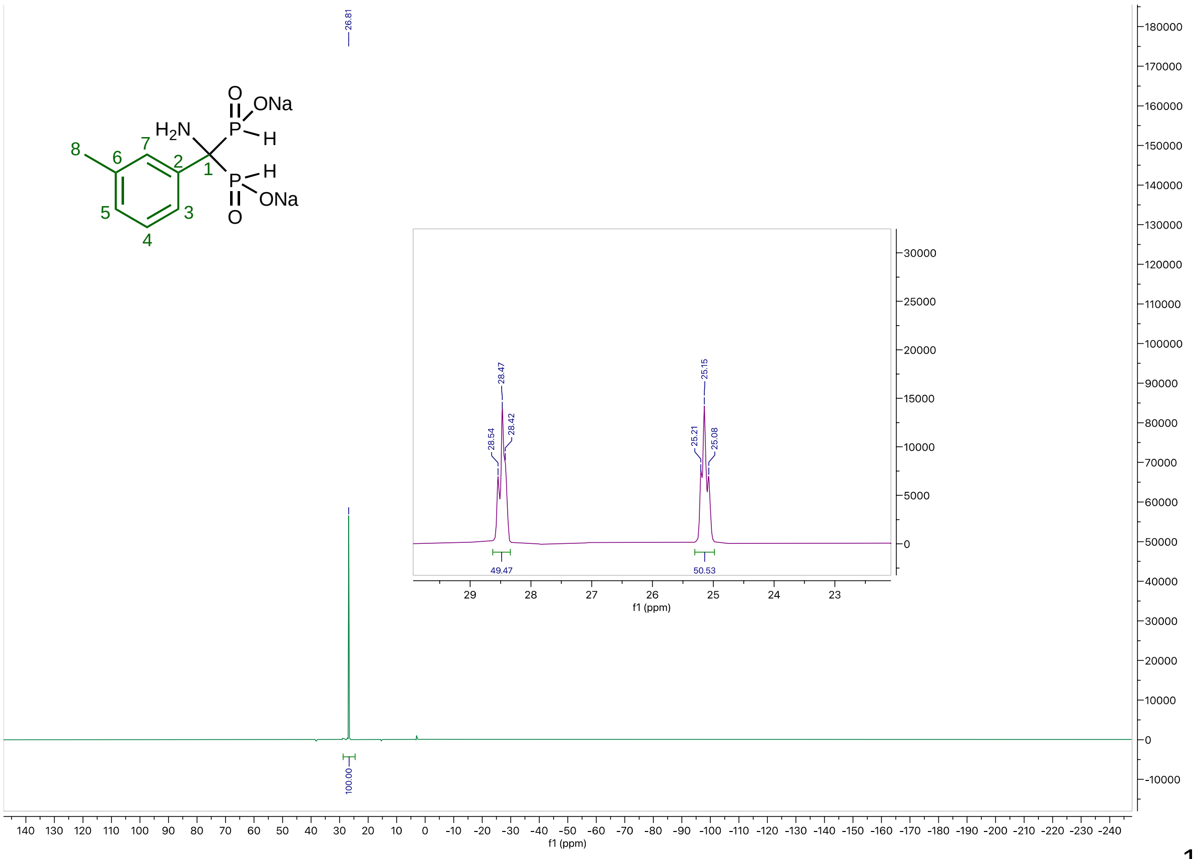

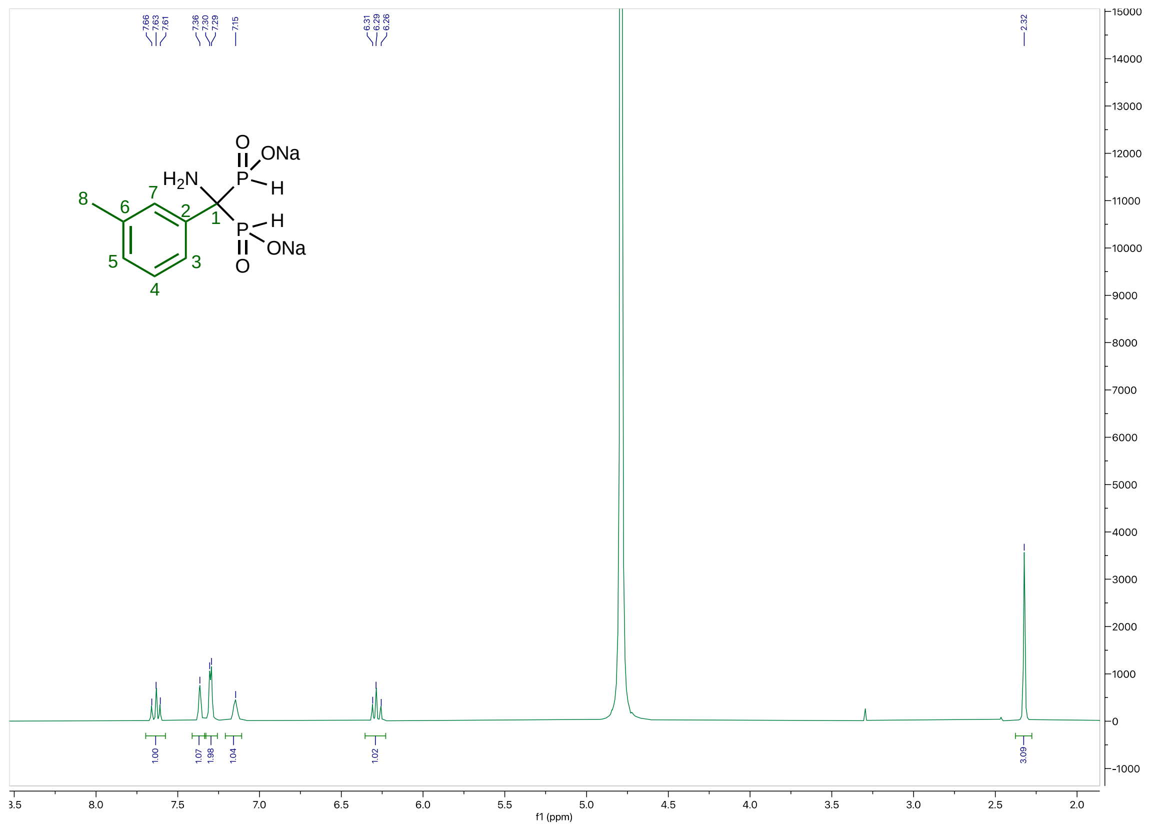

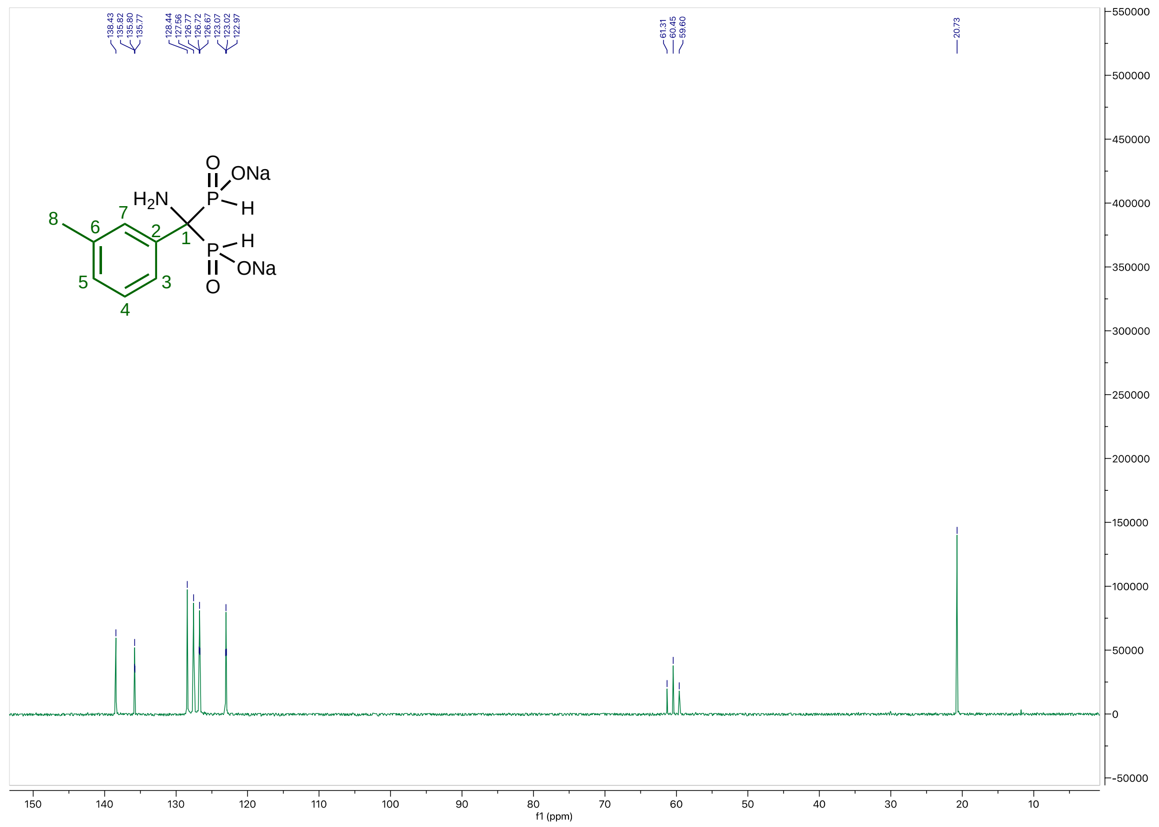

<u>Figure S8</u>. ^{31}P NMR spectrum (162 MHz, D_2O), ^{1}H NMR spectrum (400 MHz, D_2O), ^{13}C NMR (101 MHz, D_2O) of 1-amino-1-(3-tolyl)phenyl)methane-1,1-bis(H-phosphinate) disodium salts **3j**

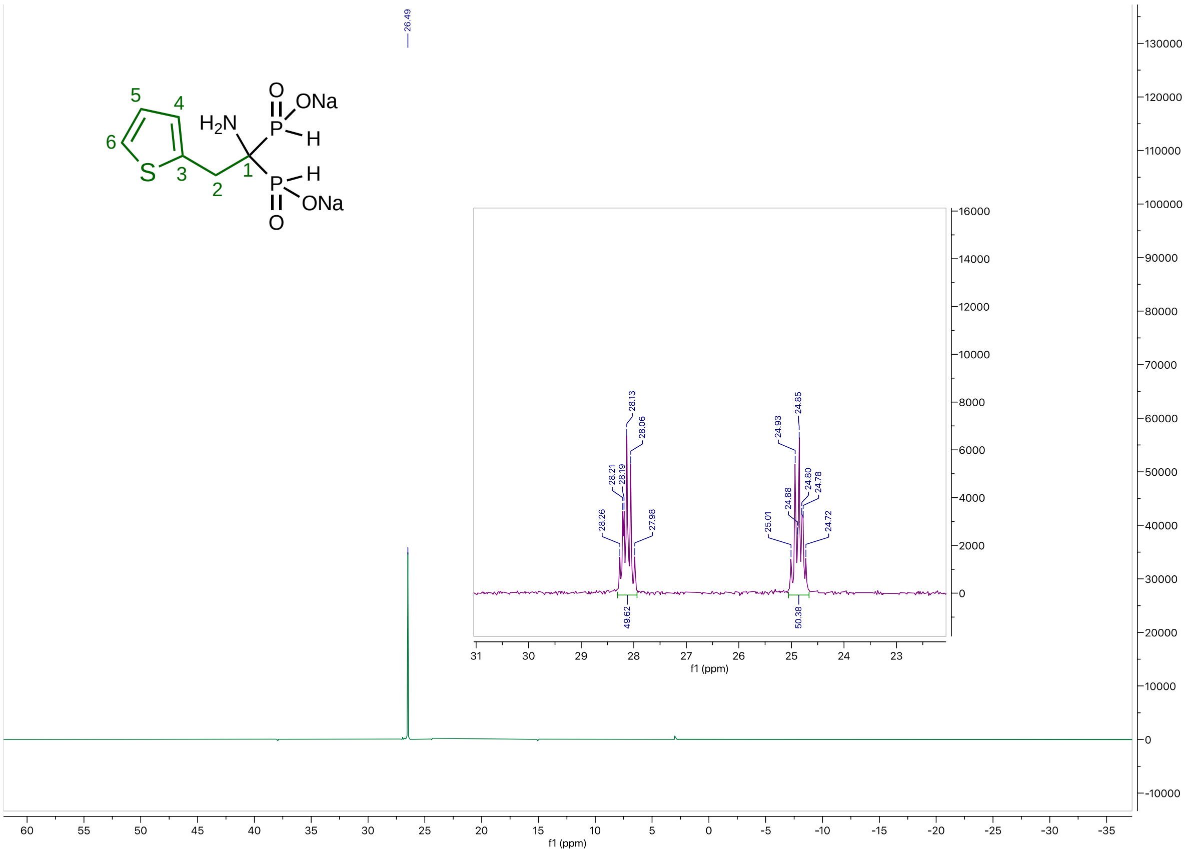

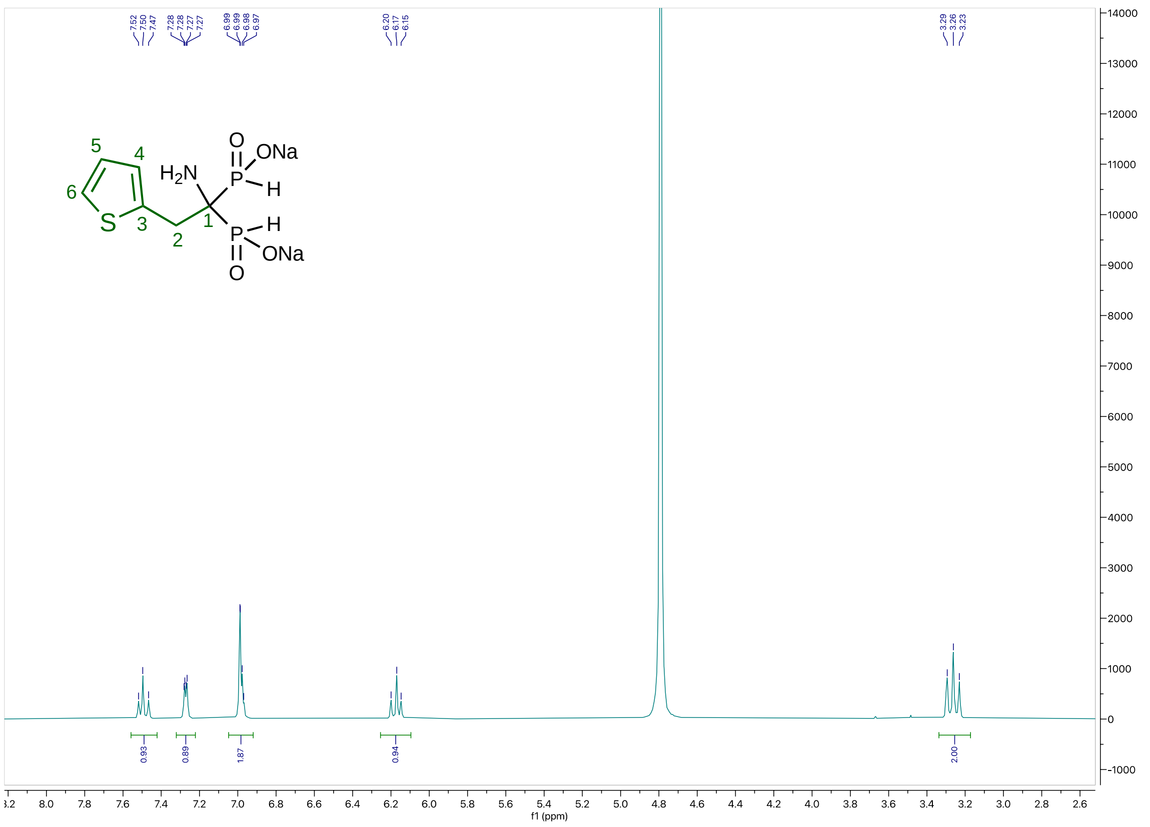

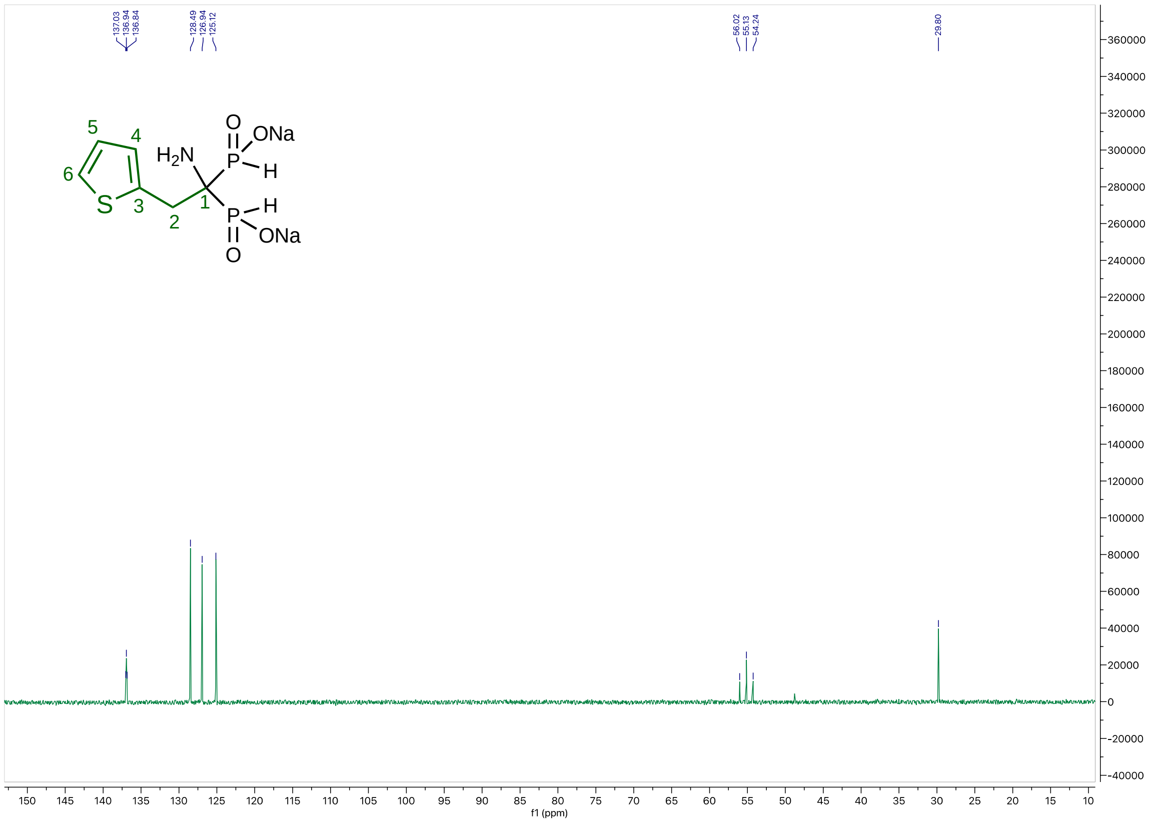

Figure S9. ^{31}P NMR spectrum (162 MHz, D_2O), ^{1}H NMR spectrum (400 MHz, D_2O), ^{13}C NMR (101 MHz, D_2O) of 1-amino-1-(2-thienyl)ethane-1,1-bis(H-phosphinate) disodium salts **3l**.
